# A MAGDM approach for evaluating the impact of artificial intelligence on education using 2-tuple linguistic *q*-rung orthopair fuzzy sets and Schweizer-Sklar weighted power average operator

**DOI:** 10.3389/frai.2024.1347626

**Published:** 2024-03-14

**Authors:** Abid Mahboob, Zafar Ullah, Ali Ovais, Muhammad Waheed Rasheed, S. A. Edalatpanah, Kainat Yasin

**Affiliations:** ^1^Department of Mathematics, Division of Science and Technology, University of Education, Lahore, Pakistan; ^2^Department of Mathematics, University of Engineering and Technology, Lahore, Pakistan; ^3^Department of Applied Mathematics, Ayandegan Institute of Higher Education, Tonekabon, Iran; ^4^Department of Mathematics, Air University Islamabad, Multan, Pakistan

**Keywords:** 2-tuple linguistic *q*-rung orthopair fuzzy set, entropy, VIKOR, impact of AI in education, MAGDM

## Abstract

The impact of artificial intelligence (AI) in education can be viewed as a multi-attribute group decision-making (MAGDM) problem, in which several stakeholders evaluate the advantages and disadvantages of AI applications in educational settings according to distinct preferences and criteria. A MAGDM framework can assist in providing transparent and logical recommendations for implementing AI in education by methodically analyzing the trade-offs and conflicts among many components, including ethical, social, pedagogical, and technical concerns. A novel development in fuzzy set theory is the 2-tuple linguistic *q*-rung orthopair fuzzy set (2TL*q*-ROFS), which is not only a generalized form but also can integrate decision-makers quantitative evaluation ideas and qualitative evaluation information. The 2TL*q*-ROF Schweizer-Sklar weighted power average operator (2TL*q*-ROFSSWPA) and the 2TL*q*-ROF Schweizer-Sklar weighted power geometric (2TL*q*-ROFSSWPG) operator are two of the aggregation operators we create in this article. We also investigate some of the unique instances and features of the proposed operators. Next, a new Entropy model is built based on 2TL*q*-ROFS, which may exploit the preferences of the decision-makers to obtain the ideal objective weights for attributes. Next, we extend the VIseKriterijumska Optimizacija I Kompromisno Resenje (VIKOR) technique to the 2TL*q*-ROF version, which provides decision-makers with a greater space to represent their decisions, while also accounting for the uncertainty inherent in human cognition. Finally, a case study of how artificial intelligence has impacted education is given to show the applicability and value of the established methodology. A comparative study is carried out to examine the benefits and improvements of the developed approach.

## 1 Introduction

AI refers to a machine's ability to carry out operations that are typically associated with human intelligence, such as learning, logical reasoning, and decision-making. AI possesses the ability to revolutionize education in a number of ways, including through enhanced assessment and feedback, personalization of the learning environment, enhancements to the teaching and learning process, and encouragement of collaboration and communication. For instance, AI can help teachers design and deliver more efficient and interesting lessons, adapt instruction to the needs and preferences of each student, give immediate and adaptive feedback, and keep track of the development and performance of their students. Additionally, AI can assist students in gaining access to a wider variety of personalized learning resources, connecting with peers and mentors around the world, and honing skills like creativity, critical thinking, and solving problems. But there are risks and challenges associated with AI as well, including those related to its impact on human roles and abilities as well as its ethical, social, and legal ramifications, data privacy and security concerns. For instance, AI might make people rethink the values and goals of education, the duties and rights of students and teachers, the ownership and use of data, and any potential biases or inequalities that might arise from using AI. Additionally, AI may have an impact on the supply and demand of human labor, the knowledge and abilities needed in the future workforce, and the ratio of human to machine intelligence. It is critical to take into account the impact of AI on education from a variety of perspectives and to ensure that its application is consistent with the principles and goals of education. Table 1 presents current research findings on the impact of AI in education.

**Table 1:** Summary of existing research on the impact of AI in education.

**References**	**Research methodology**	**Key findings**
Nguyen et al. (2023)	Thematic analysis	AI in education
Liu et al. (2023)	Fuzzy analytical hierarchy process	Long-term learning through artificial intelligence-based visual communication
Flogie and Krabonja (2023)	RAT scale	AI in education
Martinez et al. (2023)	Systematic review and meta-analysis	Effects of computing and AI on pupil achievement
Su et al. (2023)	Pedagogical approaches	Literacy using AI in early childhood education
Holmes et al. (2023)	Qualitative study	AI in education
Chen et al. (2022)	Bibliometric analysis	AI in education
Nemorin et al. (2023)	Text mining and thematic analysis	Learning and growth using AI
Prahani et al. (2022)	Bibliometric analysis	Research on AI in education during the past ten years
Shaikh et al. (2022)	Phenomenological technique	The development of a digital classroom using machine learning and artificial intelligence and its long-term effects on education will be dealt with during COVID-19
Thurzo et al. (2023)	Qualitative study	Influence of AI on learning about dentistry
Hong Yun et al. (2022)	A decision-support system	AI and machine learning in music tuition for playing games online
Teng et al. (2023)	Fuzzy comprehensive appraisal and artificial intelligence technology	The assessment and control of risks in the supply chain for sports services
Albaity et al. (2023)	Intuitionistic Fuzzy Soft WASPAS Method	The implications of AI and machine learning upon business
Paek and Kim (2021)	Linear regression model	Global research trends on the effects of AI on education

**Figure 1:**
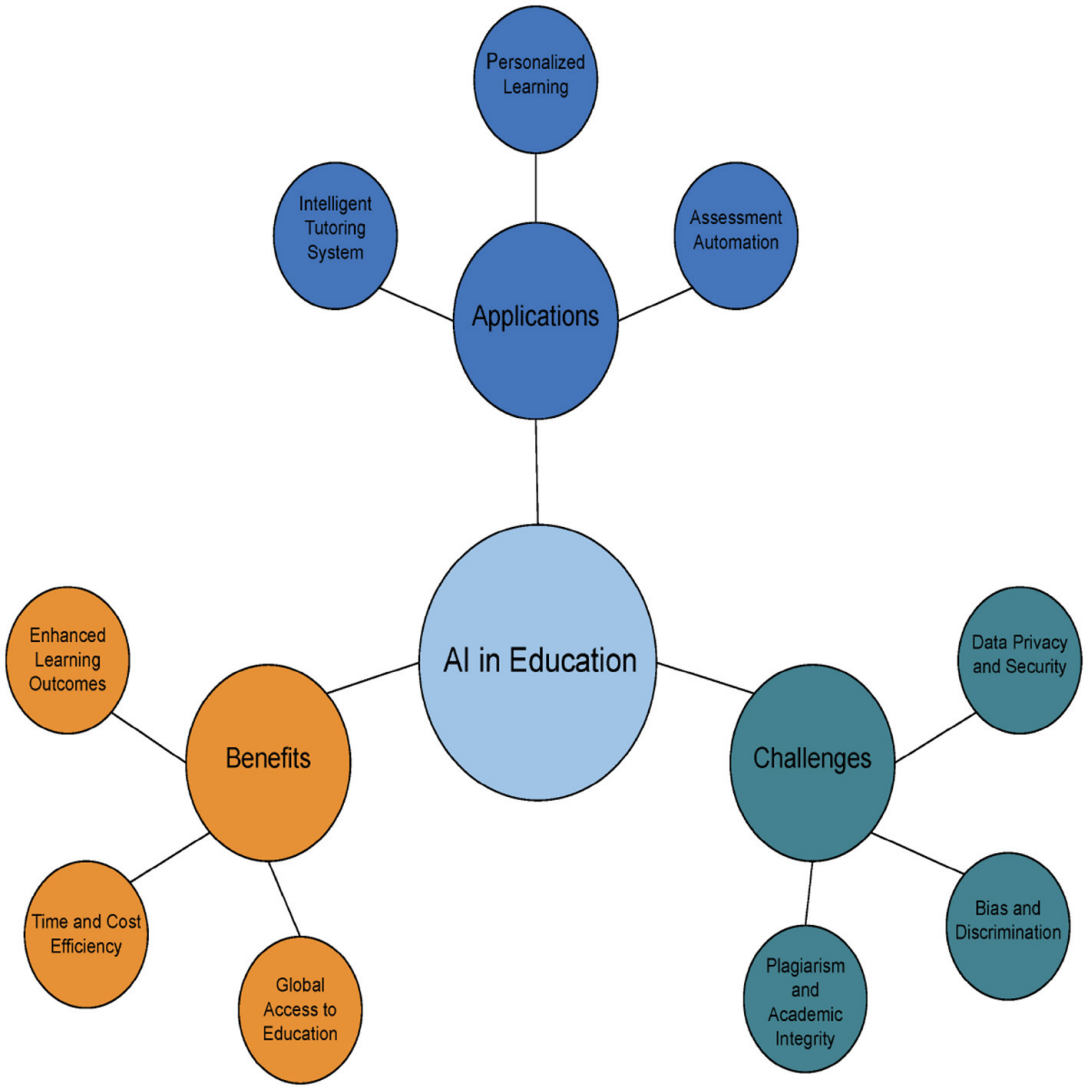
Visual representation of AI in the education.

**Figure 2:**
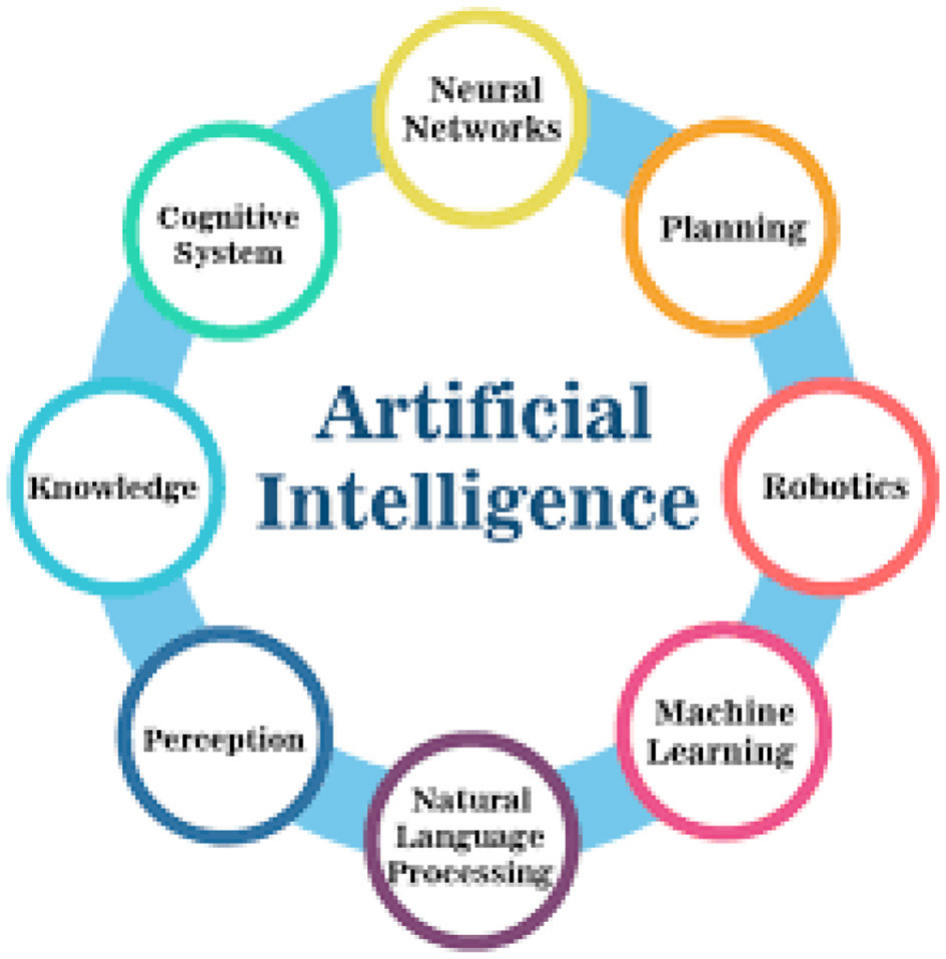
A visual of different uses of AI.

A graphical representation illustrating AI's role in education is presented in Figures 1, 2 illustrates various applications of AI across different domains.

An approach for evaluating and determining alternatives based on a variety of parameters and the preferences of decision-makers (DMs) is multi-attribute group decision-making (MAGDM). The facts and views of the group members are contradictory in MAGDM difficulties. As a result, an array of strategies and methods have been created to deal with these problems, including fuzzy logic (Zadeh, 1988), intuitionistic fuzzy logic (Takeuti and Titani, 1984), linguistic variables (Zadeh, 1983), grey numbers (Liu et al., 2012), and rough sets (Pawlak, 1982). Aggregation-based and consensus-based MAGDM techniques can be categorized into two broad groups. Aiming to combine each group member's individual preferences into a single, overall taste, aggregate-based approaches employ this preference to rank the options. Before making a conclusion, consensus-based techniques emphasize a strong agreement among the group members. The best way to choose will rely on the features and requirements of the situation because both sorts of solutions have benefits and drawbacks. MAGDM techniques may be used in a wide range of fields, including management, engineering, economics, and social sciences.

The process of MAGDM is a valuable approach for effectively handling complex and challenging data in real-world problems. MAGDM serves as a method for assigning ranking grades to limited options corresponding to distinct characteristics of various choices, playing a crucial role in the field of decision-making sciences. Practical decision-making scenarios, accurately representing these characteristic attributes is a central concern due to the intricacy and ambiguity often encountered. To deal with the issue at hand, Zadeh (1988) introduced the theory of fuzzy sets (FS), which confines membership degrees (MD) to the unit interval. The FS theory has garnered significant attention from experts and found application in diverse fields and situations (Mardani et al., 2019; Tian et al., 2022). Nevertheless, there have been instances where the FS theory has not delivered precise results. For instance, utilizing FS to handle information represented in terms of truth and falsity degrees can pose challenges. To deal with such complexity, Atanassov and Stoeva (1986) introduced the theory of intuitionistic fuzzy sets (IFS), which is a refined variation of FS designed to adeptly handle cumbersome and uncertain data. IFS encompasses both MD and non-membership degrees (NMD) through a specific set of rules 0 ≤ μ+ν ≤ 1. The theory of IFS has garnered significant research interest and has been applied in various studies (Krishankumar et al., 2023; Yue et al., 2023). Nevertheless, IFS data has a limited range and relies on the rule that mandates the total of MD and NMD to be within the unit interval, which is not always necessary. To deal with this challenge, Yager (2013) introduced the concept of Pythagorean fuzzy sets (PyFS), that represents a modified version of IFS tailored for handling complex and uncertain information. PyFS accommodates MD and NMD in a way that maintains the combined value of the squares of their pairings within the unit interval. The theory of PyFS has also garnered significant attention in a diverse range of research endeavors (Kumar and Chen, 2023; Pan et al., 2023). Nonetheless, PyFS data also has a limited range and adheres to a rule stipulating that the sum of the squares of MD and NMD should fall within the unit interval, although this restriction is not always essential. In cases where the sum of the squares of these degrees exceeds the unit interval, such as when assigning 0.7 to MD and 0.8 to NMD, PyFS conditions are violated, resulting in a value of (0.7)^2^+(0.8)^2^ = 0.49+0.64 = 1.13. To address these complex challenges, Yager (2016) introduced a modification of the PyFS theory, creating the *q*-rung orthopair fuzzy sets (*q*-ROFS) theory. This modification was aimed at improving the handling of intricate and unreliable data. *q*-ROFS encompasses both MDs and NMDs, with the *q*-powers of the pairings added restricted to the unit interval. As a result, *q*-ROFS effectively resolves issues related to such information, ensuring that the result is consistently equal to (0.7)^4^+(0.8)^4^ = 0.2401+0.4096 = 0.6497 ≤ 1. The principles governing *q*-ROFSs have undergone substantial modifications compared to established concepts like IFSs and PyFSs. These distinctive features have attracted significant attention, leading to extensive research conducted by renowned scholars and practical applications across a wide range of domains. For example, By employing transformers and *q*-ROFS theory, an innovative approach for ranking products that took into account the mass assignment of features was presented by Yin et al. (2023). Then, Fetanat and Tayebi (2023) created the *q*-ROFS based MAIRCA (Multi-Attributive Ideal-Real Comparative Analysis), a unique hybrid decision support system. Using the suggested decision support system, industrial filtration technologies were prioritized while taking sustainability and maintainability into account. Furthermore, Oraya et al. (2023) incorporated the *q*-ROFS into the computational framework to address the inconsistency and doubt of the DMs' assessments during the evaluation process. A real-world case study of residential construction projects used the novel *q*-ROF weighted influence non-linear gauge systems level-based weight assessment FlowSort approach that has been proposed. To choose the best software solution from a variety of possibilities, Wan et al. (2023) developed a novel integrated group decision-making technique for the quality assessment of the system under the interval-valued *q*-ROFSs.

The hypertension follow-up system's characteristics served as a foundation for the design of its assessment indices, which in turn represented the evaluation requirements of typical software applications and highlighted the uniqueness of the system. Additionally, For complex *q*-ROF data, Liu et al. (2023) focused on analyzing the improved Einstein operational rules. Weights were handled objectively in a decision-making technique with complex *q*-ROF information. In order to develop the collective preference data on cache placement policies with respect to the criteria, Peng X. et al. (2023) initially proposed an assessment criteria system to formulate to reflect experts' thoughts for evaluating cache placement policies. They then used the concepts of set pair analysis and *q*-ROFS to define the group preference information of cache placement policies in direct and indirect ways, respectively. Marti and Herrera (2012) created a 2-tuple linguistic (2TL) model designed to handle both verbal and nonverbal data in decision-making processes without compromising the integrity of the data. This valuable model primarily relies on symbolically translating linguistic variables and has found extensive application in various MAGDM scenarios (Rodriguez and Martinez, 2013; Romero et al., 2019). Given the complexity of objects and the inherent vagueness of human thinking, it is more appropriate to express evaluations using linguistic information rather than numerical values (Pang and Liang, 2012; Pang et al., 2023). Consequently, numerous researchers have delved into the realm of linguistic information to address MAGDM problems, devising various methods to explore linguistic representation models (Herrera and Martinez, 2000; Wu et al., 2021). Broadly speaking, the linguistic information model can be categorized into three main parts: methods based on MDs, methods utilizing linguistic symbols, and methods founded on the concept of linguistic 2-tuple. However, the first two methods possess certain limitations, as they approximate the translation process to evaluate findings in their original domain. This approximation can result in reduced precision and accuracy. In contrast, the 2TL model offers a means to enhance the accuracy of linguistic terms (LTs), computation, and interpretation of findings. Due to its recognized quality and effectiveness in handling the linguistic opinions of DMs, the 2TL model has garnered widespread adoption in decision-making scenarios, especially in addressing the transitivity issue within the context of MAGDM problems. Following the creation of the 2TL model, researchers began to identify certain limitations associated with it, specifically its capacity to handle solely uniformly distributed linguistic term sets.

To address these limitations, Wu et al. (2023) employed MAGDM techniques to develop an uncertainty model aimed at resolving the selection challenges posed by autonomous cars. They utilized the proportional hesitant 2TL term set to represent assessment information. Subsequently, Jin et al. (2023) introduced a convergent consistency improvement method, employing a minimal adjustment strategy to preserve the initial evaluation data provided by DMs while enhancing the consistency of the original 2TL preference relation to a predefined level. For 2TL Pythagorean fuzzy values, Verma and Alvarez-Miranda (2023) introduced a novel score value function and discussed its essential characteristics. Two innovative AOs, the advanced 2TL Pythagorean fuzzy weighted average operator and the advanced 2TL Pythagorean fuzzy weighted geometric operator, were proposed to combine 2TL Pythagorean fuzzy data using unique operational principles governing 2TL Pythagorean fuzzy values. In a distinctive approach, Sarwar (2023) integrated a 2TL framework with rough approximations and cloud theory, presenting a new mathematical model. Lastly, in the context of cognitive data utilized in the hospital evaluation process, Naz et al. (2023b) implemented a MAGDM approach incorporating a 2TL *T*-spherical fuzzy set.

To better capture the ambiguity of the environment and prevent information loss or distortion during the information aggregation stage, Zhang H. et al. (2023) established the concept of the interval 2-tuple *q*-ROFS. In an effort to tackle uncertainty within the realm of group decision-making, Ullah et al. (2023) recommended the utilization of MAGDM methodologies incorporating 2TL confidence level complex *q*-ROFSs. The primary aim of that research was to improve the selection process for the most appropriate recycling method. Ali et al. (2023) extended the concepts of three-way decisions and decision theoretic rough sets within the context of Complex *q*-rung orthopair 2TL variables, subsequently discussing several of its significant properties. Akram et al. (2023) investigated and expanded the evaluation and prioritization of alternatives based on the compromise solution within the framework of 2TL*q*-rung picture fuzzy sets. Pairwise comparison serves as a valuable method for DMs to articulate their preferences, particularly in situations where cognition is intricate and uncertain. Consequently, Li and Zhang (2023) opted to utilize linguistic *q*-ROF preference relations as a means to represent the cognitive information provided by experts. Additive consistency for the linguistic *q*-ROF preference relation was introduced as a mechanism to rank objects, and a consistency-focused model was built to derive the normalized linguistic *q*-ROF priority weight vector.

The Schweizer-Sklar (SS) operator is an effective method for aggregating fuzzy data with varying degrees of interdependence and relevance. This operator has the advantage of being flexible and effective at handling both prioritized and unprioritized input. Schweizer and Sklar (2011) invented the SS operator. Mahmood and ur Rehman (2023) investigated the principles for operation depend on the t-norm and t-conorm of SS and created aggregation operators based on these operational laws in the context of a Bipolar complex fuzzy BCF collection. Chen et al. (2023) proposed an advanced method for multi-attribute decision-making (MADM) in the context of *q*-rung orthopair probabilistic hesitant fuzzy environments. They introduced the *q*-rung orthopair probabilistic hesitant fuzzy SS power weighted Hamy mean operator to improve the computational capabilities of the information aggregation operator. In a similar vein, Kausar et al. (2023) not only introduced the SS aggregation operator based on cubic m-polar fuzzy sets (CmPFS) but also innovatively developed several aggregation operators using CmPFS in conjunction with the SS t-norm and t-conorm. Furthermore, Liu et al. (2023) contributed by establishing operational rules for the *q*-rung orthopair normal fuzzy set. He also extended the SS t-norm and t-conorm to *q*-rung orthopair normal fuzzy numbers. In the existing literature, there has been no exploration of the SS operator within the context of 2TL*q*-ROF information, representing a significant gap. Therefore, we introduce this operator to address this gap and demonstrate its effectiveness in handling uncertainty.

The entropy approach is used to calculate weights because it can manage data-driven weight determination in decision-making, making it appropriate for scenarios with accurate and easily accessible data. The entropy method is a widely used technique for ranking and selecting alternatives in MADM problems. The entropy method is best for situations where there is a large number of attribute, and the DMs wants to avoid bias and inconsistency in the weighting process. A novel feature selection approach was presented by Pandey et al. (2023) using intuitionistic fuzzy entropy. The amalgamated Fermatean fuzzy set and entropy approach was developed by Chang et al. (2023) to prioritize the risk of product failure items. By using a brand-new entropy measure function created specifically for this method, Aydogdu et al. (2023) produced a revolutionary TOPSIS method in a complicated spherical fuzzy context by objectively determining the weights of the criterion and DMs. Zhang Y. et al. (2023) established a stability evaluation framework for the region's rocky slopes using the entropy weighting-fuzzy theory. The usefulness and advantages of the idea of structure-based Pythagorean Fuzzy entropy that Mao et al. (2023) provide for PyFS are substantiated by numerical evidence and mathematical analysis. To enhance the currently used fuzzy entropy metric, Krishankumar et al. (2023) presented a unique entropy function for the IFS.

Opricovic (1998) suggested the VIKOR ranking system as a compromise in 1998. Zhang N. et al. (2023) suggested a VIKOR approach grounded in regret theory to tackle the MAGDM challenge with fully unknowable weight data along with Pythagorean hesitate fuzzy assessment value. Peng J. J. et al. (2023) suggested an enhanced picture fuzzy VIKOR method using bidirectional projection to carry out selection procedure. The problem of selecting environmentally friendly and sustainable suppliers was addressed by Wang et al. (2023) by integrating the TODIM and VIKOR methods, decision-making information type-2 neutrosophic numberset, and attribute weight adopted the entropy weight technique. Using the VIKOR MADM technique, Nath et al. (2023) prepared and presented a rank list of the reported catalysts. Yadav et al.'s (2023) investigation focused on the invention of the hybrid Entropy-VIKOR MCDM technique for selecting and rating dental restorative composite materials. To determine which dental composite formulation was the best, eleven performance-defining characteristics of dental composites were taken into account. Riaz et al. (2023) created a novel hybrid approach for MAGDM that combines cubic bipolar fuzzy-VIKOR method with Einstein averaging aggregation operators. Lei et al. (2023) utilized the TODIM and VIKOR techniques to address MAGDM problems as the research on the placement of medical logistics distribution centers has significant theoretical and practical application implications independently. To the greatest extent of the information we have, this would be the first ranking research study on the impact of AI in education since the VIKOR method hasn't yet been utilized for such a ranking assessment.

### 1.1 Contributions

Following are our work's essential contributions:

- In this article, we present the idea of 2TL*q*-ROFS, which combines the benefits of the 2TL set with the *q*-ROFS and has the ability to handle qualitative as well as quantitative information in a flexible and comprehensive approach.
- We created a few novel aggregation operators based on the SSWPA operator in a 2TL*q*-ROFS environment, and we looked at their unique circumstances and properties. The SSWPA operator can be reduced to some existing operators as special instances and can represent the relationships between the input arguments by utilizing a parameter vector.
- In MAGDM environments using 2TL*q*-ROFS information, we have used the Entropy-VIKOR methodology to calculate the ideal weights for the attributes and rank the outcomes. The drawbacks of subjective weighting methods and objective weighting methods can be overcome by the Entropy-VIKOR methodology, which can produce a ranking result that is more reasonable and reliable.
- Using an actual case to look at whether the impact of AI in education, we have demonstrated the applicability and efficacy of our proposed method. We also contrasted our approach with a few already in use.

### 1.2 The suggested study's architecture

The remainder of the paper will be organized according to the following format: In Section 2, we will provide a concise overview of fundamental definitions and concepts related to 2TL*q*-ROFS and its associated operational laws. Section 3 will delve into SS power average and SS power geometric operators, along with their weighted variants tailored for 2TL*q*-ROF numbers. We will also explore their respective properties. The 2TL*q*-ROF-Entropy-VIKOR method for solving the MAGDM problem will be presented in Section 4. In Section 5, we will demonstrate the effectiveness and feasibility of our proposed model through a practical case study focused on the impact of artificial intelligence in education. Additionally, we will conduct a comparative analysis between our approach and prior research. Lastly, Section 6 will conclude the paper, highlighting key findings, acknowledging limitations, and outlining potential directions for future research.

## 2 Preliminaries

In this section, we provide a recap of important concepts, such as 2TL*q*-ROFS, to enhance the clarity and comprehension of the subsequent sections.

** Definition 1**. Naz et al. (2022b). Consider a set *S* defined as follows: *S* = {ℜ_𝔩_|𝔩 = 0, …, τ}. This set is characterized as a LTS with an odd cardinality. If $\left({ℜ}_{r}(\partial ),§(\partial )),({ℜ}_{t}(\partial ),G(\partial )\right)$ is defined for ${ℜ}_{r}(\partial ),{ℜ}_{t}(\partial )\in S,\;§(\partial ),G(\partial )\in [-0.5,0.5)$, where $\left({ℜ}_{r}(\partial ),§(\partial )\right)$ and $\left({ℜ}_{t}(\partial ),G(\partial )\right)$ represent the MD and NMD by 2TL terms, respectively. Equation (1) defines the 2TL*q*-ROFS:


(1) $$W=\{\langle \partial ,(({ℜ}_{r}(\partial ),§(\partial )),({ℜ}_{t}(\partial ),G(\partial )))\rangle |\partial \in L\},$$

where $0\leq \Delta^{-1}({ℜ}_{r}(\partial ),§(\partial ))\leq \tau ,0\leq \Delta^{-1}({ℜ}_{t}(\partial ),G(\partial ))\leq \tau ,$ and $0\leq {\left(\Delta^{-1}({ℜ}_{r}(\partial ),§(\partial ))\right)}^{q}+{\left(\Delta^{-1}({ℜ}_{t}(\partial ),G(\partial ))\right)}^{q}\leq \tau^{q}.$

The descriptions that follow of the score function and accuracy function can be utilized when comparing any two 2TL*q*-ROFNs:

** Definition 2**. Naz et al. (2022b). Assume that $W=(({ℜ}_{r},§),({ℜ}_{t},G))$ corresponds to 2TL*q*-ROFN. Consequently, the Equation (2) can be utilized for presenting the score function of a 2TL*q*-ROFN:


(2) $$\begin{array}{l} Ϝ(W)=\Delta \left(\frac{\tau }{2}\left(1+{\left(\frac{\Delta^{-1}({ℜ}_{r},§)}{\tau }\right)}^{q}-{\left(\frac{\Delta^{-1}({ℜ}_{t},G)}{\tau }\right)}^{q}\right)\right),\\ \;\Delta^{-1}(Ϝ(W))\in [0,\tau ], \end{array}$$

Additionally, the accuracy function ℷ is represented by Equation (3).


(3) $$\begin{array}{l} ℷ(W)=\Delta \left(\tau \left({\left(\frac{\Delta^{-1}({ℜ}_{r},§)}{\tau }\right)}^{q}+{\left(\frac{\Delta^{-1}({ℜ}_{t},G)}{\tau }\right)}^{q}\right)\right),\\ \;\Delta^{-1}(ℷ(W))\in [0,\tau ]. \end{array}$$

** Definition 3**. Naz et al. (2022b). Consider $W_{1}=(({ℜ}_{r_{1}},{§}_{1}),({ℜ}_{t_{1}},G_{1}))$ and $W_{2}=(({ℜ}_{r_{2}},{§}_{2}),({ℜ}_{t_{2}},G_{2}))$ are two 2TL*q*-ROFNs, then these two 2TL*q*-ROFNs can be compared using the rules listed below:

(1) If $Ϝ(W_{1})>Ϝ(W_{2})$, then $W_{1}≻W_{2}$;
(2) If $Ϝ(W_{1})<Ϝ(W_{2})$, then $W_{1}≺W_{2}$;
(3) If $Ϝ(W_{1})=Ϝ(W_{2})$, then
  - If $ℷ\left(W_{1}\right)>ℷ\left(W_{2}\right)$, then $W_{1}≻W_{2}$;
  - If $ℷ\left(W_{1}\right)<ℷ\left(W_{2}\right)$, then $W_{1}≺W_{2}$;
  - If $ℷ\left(W_{1}\right)=ℷ\left(W_{2}\right)$, then $W_{1}\sim W_{2}$.

Based on algebraic operations, the following operational laws for 2TL*q*-ROFNs can be established:

**Definition 4**. Naz et al. (2022b). Let $W=(({ℜ}_{r},§),({ℜ}_{t},G))$, $W_{1}=(({ℜ}_{r_{1}},{§}_{1}),({ℜ}_{t_{1}},G_{1}))$ and $W_{2}=(({ℜ}_{r_{2}},{§}_{2}),({ℜ}_{t_{2}},G_{2}))$ are three 2TL*q*-ROFNs, whereby *q*≥1, afterwards


$$\begin{array}{l} 1.W_{1}⊕W_{2}=\left(\begin{array}{l} \Delta \left(\tau \sqrt[{q}]{1-\left(1-{\left(\frac{\Delta^{-1}({ℜ}_{r_{1}},{§}_{1})}{\tau }\right)}^{q}\right)\left(1-{\left(\frac{\Delta^{-1}({ℜ}_{r_{2}},{§}_{2})}{\tau }\right)}^{q}\right)}\right),\\ \Delta \left(\tau \left(\frac{\Delta^{-1}({ℜ}_{t_{1}},G_{1})}{\tau }\right)\left(\frac{\Delta^{-1}({ℜ}_{t_{2}},G_{2})}{\tau }\right)\right) \end{array}\right);\\ 2.W_{1}⊗W_{2}=\left(\begin{array}{l} \Delta \left(\tau \left(\frac{\Delta^{-1}({ℜ}_{r_{1}},{§}_{1})}{\tau }\right)\left(\frac{\Delta^{-1}({ℜ}_{r_{2}},{§}_{2})}{\tau }\right)\right),\\ \Delta \left(\tau \sqrt[{q}]{1-\left(1-{\left(\frac{\Delta^{-1}({ℜ}_{t_{1}},G_{1})}{\tau }\right)}^{q}\right)\left(1-{\left(\frac{\Delta^{-1}({ℜ}_{t_{2}},G_{2})}{\tau }\right)}^{q}\right)}\right) \end{array}\right);\\ 3.\;\lambda W=\left(\begin{array}{l} \Delta \left(\tau \sqrt[{q}]{1-{\left(1-{\left(\frac{\Delta^{-1}({ℜ}_{r},§)}{\tau }\right)}^{q}\right)}^{\lambda }}\right),\Delta \left(\tau {\left(\frac{\Delta^{-1}({ℜ}_{t},G)}{\tau }\right)}^{\lambda }\right) \end{array}\right),\;\lambda >0;\\ 4.\;W^{\lambda }=\left(\begin{array}{l} \Delta \left(\tau {\left(\frac{\Delta^{-1}({ℜ}_{r},§)}{\tau }\right)}^{\lambda }\right),\Delta \left(\tau \sqrt[{q}]{1-{\left(1-{\left(\frac{\Delta^{-1}({ℜ}_{t},G)}{\tau }\right)}^{q}\right)}^{\lambda }}\right) \end{array}\right),\;\lambda >0. \end{array}$$

**Definition 5**. Naz et al. (2022b). Let $W_{1}=(({ℜ}_{r_{1}},{§}_{1}),({ℜ}_{t_{1}},G_{1}))$ and $W_{2}=(({ℜ}_{r_{2}},{§}_{2}),({ℜ}_{t_{2}},G_{2}))$, respectively, two 2TL*q*-ROFNs. Following the subsequent description, the normalized 2TL*q*-ROF Hamming distance is:


(4) $$d(W_{1},W_{2})=\Delta \left(\frac{\tau }{2}\left(\begin{array}{l} \left|{\left(\frac{\Delta^{-1}({ℜ}_{r_{1}},{§}_{1})}{\tau }\right)}^{q}-{\left(\frac{\Delta^{-1}({ℜ}_{r_{2}},{§}_{2})}{\tau }\right)}^{q}\right|\\ +\left|{\left(\frac{\Delta^{-1}({ℜ}_{t_{1}},G_{1})}{\tau }\right)}^{q}-{\left(\frac{\Delta^{-1}({ℜ}_{t_{2}},G_{2})}{\tau }\right)}^{q}\right| \end{array}\right)\right).$$

## 3 The 2TL*q*-ROFSSWPA operator

** Definition 6**. Yager (2001). The power average (PA) operator, a non-linear weighted average aggregation operator was proposed by Yager and described as follows Equation (5)


(5) $$PA(r_{1},r_{2},\ldots ,r_{ň})=\frac{\sum_{\psi =1}^{ň}(1+ℸ(r_{\psi }))r_{\psi }}{\sum_{\psi =1}^{ň}(1+ℸ(r_{\psi }))},$$

where $ℸ(r_{\psi })=\sum_{\phi =1,\phi \neq \psi }^{ň}ℷ(r_{\psi },r_{\phi })$ and ℷ(r,⊔) is the support for 𝔯 and ⊔ which meets the three prerequisites listed below:

-
ℷ(r,⊔)∈[0,1];

-
ℷ(r,⊔)=ℷ(t,r);

- ℷ(r,⊔)≥ℷ(a,b), if|r−t|<|a−b|..

**Definition 7**. Let $W_{\phi }=(({ℜ}_{r_{\phi }},{§}_{\phi }),({ℜ}_{t_{\phi }},G_{\phi }))(\phi =1,2,\ldots ,\overset{ˇ}{n})$ be a group of 2TL*q*-ROFNs. The 2TL*q*-ROFSSWPA operator serves as a mapping *T*^ň^→*T* as shown in Equation (6).


(6) $$\text{2TL}q-\text{ROFSSWPA}(W_{1},W_{2},\ldots ,W_{ň})=\overset{ň}{\underset{\phi =1}{⊕}}{ð}_{\phi }W_{\phi },$$

in which *T* is the set of 2TL*q*-ROFNs, $\beta ={\left(\beta_{1},\beta_{2},\ldots ,\beta_{ň}\right)}^{T}$ is the weight vector of $W_{\phi }\left(\phi =1,2,\ldots ,ň\right)$, such that β_ϕ_∈[0, 1] and $\overset{ň}{\underset{\phi =1}{\sum }}\beta_{\phi }=1.$

In this case, *ð*_ϕ_ might be determined as follows:

**Step 1.** Determine the support degree among $W^{\phi }$ and $W^{\psi }\left(\phi =1,2,\ldots ,ň,;\phi \neq \psi \right)$ by using Equation (7).

(7) $$\begin{array}{l} ℸ\left(W^{\phi },W^{\psi }\right)=1-d\left(W^{\phi },W^{\psi }\right). \end{array}$$

Wherein the $d\left(W^{\phi },W^{\psi }\right)$ is the distance amongst $W^{\phi }$ and $W^{\psi }$.
**Step 2.** Conduct the syntheses support degree calculations $ℶ\left(W^{\phi }\right)$ of $\left(W^{\phi }\right)$ by using Equation (8).

(8) $$\begin{array}{l} ℶ\left(W^{\phi }\right)=\overset{ň}{\underset{\phi =1,\phi \neq \psi }{\sum }}\beta_{\phi }ℸ\left(W^{\phi },W^{\psi }\right) \end{array}$$

**Step 3.** Calculate the complete set of power weights using Equation (9):

(9) $$\begin{array}{l} {ð}_{\phi }=\frac{\beta_{\phi }\left(1+ℶ\left(W^{\phi }\right)\right)}{\overset{ň}{\underset{\phi =1}{\sum }}\beta_{\phi }\left(1+ℶ\left(W^{\phi }\right)\right)}. \end{array}$$

Theorem 1. Let $W_{\phi }=(({ℜ}_{r_{\phi }},{§}_{\phi }),({ℜ}_{t_{\phi }},G_{\phi }))(\phi =1,2,\ldots ,\overset{ˇ}{n})$ a group of 2TL*q*-ROFNs with a weight vector $\beta ={\left(\beta_{1},\beta_{2},\ldots ,\beta_{ň}\right)}^{T}$, such that β_ϕ_∈[0, 1] and $\overset{ň}{\underset{\phi =1}{\sum }}\beta_{\phi }=1$, the 2TL_*q*_-ROFSSWPA operator is defined as shown in Equation (10).


(10) $$\begin{array}{l} \text{2TL}q-\text{ROFSSWPA}(W_{1},W_{2},\ldots ,W_{ň})\\ =\left(\begin{array}{l} \Delta \left(\tau {\left(1-{\left(\sum_{\phi =1}^{ň}{ð}_{\phi }{\left(1-{\left(\frac{\Delta^{-1}({ℜ}_{r_{\phi }},{§}_{\phi })}{\tau }\right)}^{q}\right)}^{\xi }\right)}^{\frac{1}{\xi }}\right)}^{\frac{1}{q}}\right),\\ \Delta \left(\tau {\left(\sum_{\phi =1}^{ň}{ð}_{\phi }{\left(\frac{\Delta^{-1}({ℜ}_{t_{\phi }},G_{\phi })}{\tau }\right)}^{q\xi }\right)}^{\frac{1}{q\xi }}\right) \end{array}\right). \end{array}$$

*Proof*. By utilizing the mathematical induction technique to the positive integer *ň*, we demonstrate that the Equation (10) holds.

(a) Whenever *ň* = 1, afterwards

$${ð}_{1}W_{1}=\left(\text{​​}\begin{array}{l} \Delta \left(\tau {\left(1-{\left({ð}_{1}{\left(1-{\left(\frac{\Delta^{-1}({ℜ}_{r_{1}},{§}_{1})}{\tau }\right)}^{q}\right)}^{\xi }\right)}^{\frac{1}{\xi }}\text{​​}\right)}^{\frac{1}{q}}\text{​​}\right),\\ \Delta \left(\tau {\left({ð}_{1}{\left(\frac{\Delta^{-1}({ℜ}_{t_{1}},G_{1})}{\tau }\text{​​}\right)}^{q\xi }\text{​​}\right)}^{\frac{1}{q\xi }}\text{​​}\right) \end{array}\text{​​}\right).$$

Thus, Equation (10) holds for *ň* = 1.
(b) Assume that Equation (10) holds for $\overset{ˇ}{n}=\overset{ˇ}{m}$,

$$\text{2TL}q\text{-ROFSSWPA}(W_{1},W_{2},\ldots ,W_{\phi })=\left(\begin{array}{l} \Delta \left(\tau {\left(1-{\left(\sum_{\phi =1}^{\overset{ˇ}{n}}{ð}_{\phi }{\left(1-{\left(\frac{\Delta^{-1}({ℜ}_{r_{\phi }},{§}_{\phi })}{\tau }\right)}^{q}\right)}^{\xi }\right)}^{\frac{1}{\xi }}\right)}^{\frac{1}{q}}\right),\\ \Delta \left(\tau {\left(\sum_{\phi =1}^{\overset{ˇ}{m}}{ð}_{\phi }{\left(\frac{\Delta^{-1}({ℜ}_{t_{\phi }},G_{\phi })}{\tau }\right)}^{q\xi }\right)}^{\frac{1}{q\xi }}\right) \end{array}\right).$$

Then, by inductive assumption, when $\overset{ˇ}{n}=\overset{ˇ}{m}+1$

$$\begin{array}{l} \text{2TL}q\text{-ROFSSWPA}(W_{1},W_{2},\ldots ,W_{\overset{ˇ}{m}},W_{\overset{ˇ}{m}+1})\\ =\text{2TL}q\text{-ROFSSWPA}(W_{1},W_{2},\ldots ,W_{\overset{ˇ}{m}})⊕{ð}_{\overset{ˇ}{m}+1}W_{\overset{ˇ}{m}+1}\\ =\left(\begin{array}{l} \Delta \left(\tau {\left(1-{\left(\sum_{\phi =1}^{\overset{ˇ}{m}}{ð}_{\phi }{\left(1-{\left(\frac{\Delta^{-1}({ℜ}_{r_{\phi }},{§}_{\phi })}{\tau }\right)}^{q}\right)}^{\xi }\right)}^{\frac{1}{\xi }}\right)}^{\frac{1}{q}}\right),\\ \Delta \left(\tau {\left(\sum_{\phi =1}^{\overset{ˇ}{m}}{ð}_{\phi }{\left(\frac{\Delta^{-1}({ℜ}_{t_{\phi }},G_{\phi })}{\tau }\text{​​}\right)}^{q\xi }\text{​​}\right)}^{\frac{1}{q\xi }}\text{​​}\right) \end{array}\text{​​}\right)\\ ⊕\left(\text{​​}\begin{array}{l} \Delta \left(\text{​​}\tau {\left(\text{​​}1-{\left(\text{​​}{ð}_{\overset{ˇ}{m}+1}{\left(\text{​​}1-{\left(\text{​​}\frac{\Delta^{-1}({ℜ}_{r_{\overset{ˇ}{m}+1}},{§}_{\overset{ˇ}{m}+1})}{\tau \text{​​}}\right)}^{q}\text{​​}\right)}^{\xi }\text{​​}\right)}^{\frac{1}{\xi }}\right)}^{\frac{1}{q}}\right),\\ \Delta \left(\tau {\left({ð}_{\overset{ˇ}{m}+1}{\left(\text{​​}\frac{\Delta^{-1}({ℜ}_{t_{\overset{ˇ}{m}+1}},G_{\overset{ˇ}{m}+1})}{\tau }\right)}^{q\xi }\text{​​}\right)}^{\frac{1}{q\xi }}\text{​​}\right) \end{array}\text{​​}\right). \end{array}$$

$$=\left(\text{​​}\begin{array}{l} \Delta \left(\text{​​}\tau {\left(\text{​​}1-{\left(\sum_{\phi =1}^{\overset{ˇ}{m}+1}{ð}_{\phi }{\left(1-{\left(\frac{\Delta^{-1}({ℜ}_{r_{\phi }},{§}_{\phi })}{\tau }\right)}^{q}\right)}^{\xi }\text{​​}\right)}^{\frac{1}{\xi }}\right)}^{\frac{1}{q}}\text{​​}\right),\\ \Delta \left(\tau {\left(\sum_{\phi =1}^{\overset{ˇ}{m}+1}{ð}_{\phi }{\left(\frac{\Delta^{-1}({ℜ}_{t_{\phi }},G_{\phi })}{\tau }\right)}^{q\xi }\right)}^{\frac{1}{q\xi }}\right) \end{array}\text{​​}\right).$$

Consequently, it can be concluded that Equation (10) holds for a positive integer $\overset{ˇ}{n}=\overset{ˇ}{m}+1$. Although Equation (10) holds for every ň ≥ 1, it can be proved using the mathematical induction method.

          ☐

Theorem 2. Let $W_{\phi }=(({ℜ}_{r_{\phi }},{§}_{\phi }),({ℜ}_{t_{\phi }},G_{\phi }))$ and ${W^{\prime }}_{\phi }=(({ℜ}_{r_{\phi }{\;}^{\prime }},{§}_{\phi^{\prime }}),({ℜ}_{t_{\phi }{\;}^{\prime }},G_{\phi^{\prime }}))(\phi =1,2,\ldots ,\overset{ˇ}{n})$ be two sets of 2TL*q*-ROFNs, the 2TL*q*-ROFSSWPA operator satisfies the following characteristics:

1. (Idempotency) If all $W_{\phi }=(({ℜ}_{r_{\phi }},{§}_{\phi }),({ℜ}_{t_{\phi }},G_{\phi }))(\phi =1,2,\ldots ,\overset{ˇ}{n})$ are identical with regard to each ϕ, then

$$\begin{array}{l} \text{2TL}q\text{-ROFSSWPA}\left(W_{1},W_{2},\ldots ,W_{ň}\right)=W. \end{array}$$

2. (Monotonicity) If $W_{\phi }\leq W_{\phi }^{\prime }$, for all *ϕ*, then

$$\begin{array}{l} \text{2TL}q\text{-ROFSSWPA}\left(W_{1},W_{2},\ldots ,W_{ň}\right)\\ \leq \text{2TL}q\text{-ROFSSWPA}\left(W_{1}^{\prime },W_{2}^{\prime },\ldots ,W_{ň}^{\prime }\right). \end{array}$$

3. (Boundedness) Let $W_{\phi }=(({ℜ}_{r_{\phi }},{§}_{\phi }),({ℜ}_{t_{\phi }},G_{\phi }))(\phi =1,2,\ldots ,\overset{ˇ}{n})$ be a group of 2TL*q*-ROFNs, and consider $W^{-}=(\underset{\phi }{\min}({ℜ}_{r_{\phi }},{§}_{\phi }),\underset{\phi }{\max}({ℜ}_{t_{\phi }},G_{\phi }))$ and $W^{+}=(\underset{\phi }{\min}({ℜ}_{r_{\phi }},{§}_{\phi }),\underset{\phi }{\max}({ℜ}_{t_{\phi }},G_{\phi }))$, then

$$\begin{array}{l} W^{-}\leq \text{2TL}q\text{-ROFSSWPA}\left(W_{1},W_{2},\ldots ,W_{ň}\right)\leq W^{+}. \end{array}$$

**Definition 8**. Xu and Yager (2009). Building on the power average operator (PA) and the geometric mean, Xu and Yager suggested a new operator termed the power geometric operator (PG), defined in Equation (11).


(11) $$PG(r_{1},r_{2},\ldots ,r_{ň})=\sum_{\psi =1}^{ň}r_{\psi }^{\frac{1+ℶ(r_{\psi })}{\sum_{\psi =1}^{n}(1+ℶ(r_{\psi })}}.$$

The non-linear weighted aggregation tools PA and PG utilize weighting vectors that depend upon input values. These operators permit values to be aggregated in a manner that complements and strengthens one another. In other words, when the values depicted by 𝔯_ψ_ and 𝔯_ϕ_ are near together, they are more identical and lend more powerful suppor to one another. Several 2TL*q*-ROFSSWPG aggregation operators are defined based on the PG and the 2TL*q*-ROFSSWPA operator.

**Definition 9**. Let $W_{\phi }=(({ℜ}_{r_{\phi }},{§}_{\phi }),({ℜ}_{t_{\phi }},G_{\phi }))(\phi =1,2,\ldots ,\overset{ˇ}{n})$ be a group of 2TL*q*-ROFNs. The 2TL*q*-ROFSSWPG operator serves as a mapping *T*^ň^→*T* as shown in Equation (12).


(12) $$\text{2TL}q\text{-ROFSSWPG}(W_{1},W_{2},\ldots ,W_{\overset{ˇ}{n}})=\overset{ň}{\underset{\phi =1}{⊗}}W_{\phi }^{{ð}_{\phi }},$$

in which *T* is the set of 2TL*q*-ROFNs, $\beta ={\left(\beta_{1},\beta_{2},\ldots ,\beta_{ň}\right)}^{T}$ is the weight vector of $W_{\phi }\left(\phi =1,2,\ldots ,ň\right)$, such that β_ϕ_ ∈ [0, 1] and $\overset{ň}{\underset{\phi =1}{\sum }}\beta_{\phi }=1.$

Where *ð*_ϕ_ might be determined as:

**Step 1.** Determine the support degree among $W^{\phi }$ and $W^{\psi }\left(\phi =1,2,\ldots ,ň,;\phi 6=\psi \right)$ by using Equation (13).

(13) $$\begin{array}{l} ℸ\left(W^{\phi },W^{\psi }\right)=1-d\left(W^{\phi },W^{\psi }\right). \end{array}$$

Wherein the $d\left(W^{\phi },W^{\psi }\right)$ is the distance amongst $W^{\phi }$ and $W^{\psi }$.
**Step 2.** Conduct the syntheses support degree calculations $ℶ\left(W^{\phi }\right)$ of $\left(W^{\phi }\right)$ by using Equation (14).

(14) $$\begin{array}{l} ℶ\left(W^{\phi }\right)=\overset{ň}{\underset{\phi =1,\phi \neq \psi }{\sum }}\beta_{\phi }ℸ\left(W^{\phi },W^{\psi }\right) \end{array}$$

**Step 3.** Calculate the complete set of power weights using Equation (15)

(15) $$\begin{array}{l} {ð}_{\phi }=\frac{\beta_{\phi }\left(1+ℶ\left(W^{\phi }\right)\right)}{\overset{ň}{\underset{\phi =1}{\sum }}\beta_{\phi }\left(1+ℶ\left(W^{\phi }\right)\right)}. \end{array}$$

Theorem 3. Let $W_{\phi }=(({ℜ}_{r_{\phi }},{§}_{\phi }),({ℜ}_{t_{\phi }},G_{\phi }))(\phi =1,2,\ldots ,\overset{ˇ}{n})$ be a group of 2TL*q*-ROFNs containing an appropriate weight vector $\beta ={\left(\beta_{1},\beta_{2},\ldots ,\beta_{ň}\right)}^{T}$, such that β_ϕ_ ∈ [0, 1] and $\overset{ň}{\underset{\phi =1}{\sum }}\beta_{\phi }=1$. Consequently, the operator's 2TL*q*-ROFSSWPG's aggregate value stays a 2TL*q*-ROFN, the 2TL*q*-ROFSSWPA operator is defined as shown in Equation (16).

(16) $$\begin{array}{l} \text{2TL}q\text{-ROFSSWPG}(W_{1},W_{2},\ldots ,W_{ň})\\ =\left(\begin{array}{l} \Delta \left(\tau {\left(\sum_{\phi =1}^{ň}{ð}_{\phi }{\left(\frac{\Delta^{-1}({ℜ}_{r_{\phi }},{§}_{\phi })}{\tau }\right)}^{q\xi }\right)}^{\frac{1}{q\xi }}\right),\\ \Delta \left(\tau {\left(1-{\left(\sum_{\phi =1}^{ň}{ð}_{\phi }{\left(1-{\left(\frac{\Delta^{-1}({ℜ}_{t_{\phi }},G_{\phi })}{\tau }\right)}^{q}\right)}^{\xi }\right)}^{\frac{1}{q}}\right)}^{\frac{1}{\xi }}\right) \end{array}\right). \end{array}$$

*Proof*. The proof resembles that of the Theorem 1.       ☐
  The idempotency, monotonicity, and boundedness of the 2TL*q*-ROFSSWPA operator are also characteristics of the 2TL*q*-ROFSSWPG operator.

## 4 Decision analysis with entropy-VIKOR approach in MAGDM environment

The framework described in this section combines the VIKOR and Entropy methods in the 2TL*q*-ROF environment. The VIKOR and Entropy methods are important in the context of MAGDM. The 2TL*q*-ROF-VIKOR methodology, from which the weight is obtained via the Entropy method, is explained in detail as follows:

**Step 1** Construct the 2TL*q*-ROF decision matrix. Consider there are a variety of ‘$\overset{ˇ}{m}$' alternatives. $\Theta =\left\{\Theta_{1},\Theta_{2},\ldots ,\Theta_{\overset{ˇ}{m}}\right\}$ and a set of ‘*ň*' attributes Ω = {Ω_1_, Ω_2_, …, Ω_*ň*_}. A group of DMs Φ = {Φ_1_, Φ_2_, …, Φ_𝔢_} is created with the weight vector $\beta^{\prime }={\left(\beta_{1}^{\prime },\beta_{2}^{\prime },\ldots ,\beta_{𝔢}^{\prime }\right)}^{T}$ where *β*′ ∈ [0, 1] and $\sum_{\gamma =1}^{e}{\beta^{\prime }}_{\gamma }=1$, to articulate their views regarding each alternative Θ_*ψ*_ about the attribute Ω_*ϕ*_ in the terms of 2TL*q*-ROFNs. Assuming that each DM Φ_*γ*_ presents information about the assessment in terms of the 2TL*q*-ROF decision matrix $\hbar^{\gamma }={\left[W_{\psi \phi }^{\gamma }\right]}_{\overset{ˇ}{m}\times \overset{ˇ}{n}}=(({ℜ}_{r_{\psi \phi }}^{\gamma },{§}_{\psi \phi }^{\gamma }),({ℜ}_{t_{\psi \phi }}^{\gamma },c_{\psi \phi }^{\gamma }))(\psi =1,2,\ldots ,\overset{ˇ}{m},\phi =1,2,\ldots ,\overset{ˇ}{n})$ as:

(17) $$\begin{array}{l} \hbar^{\gamma }=[W_{\psi \phi }^{\gamma }{]}_{\overset{ˇ}{m}\times \overset{ˇ}{n}}\\ \;={\left[\begin{array}{cccc} \left({\left({ℜ}_{r_{11}},{§}_{11}\right)}^{\gamma },{\left({ℜ}_{t_{11}},G_{11}\right)}^{\gamma }\right) & \left({\left({ℜ}_{r_{12}},{§}_{12}\right)}^{\gamma },{\left({ℜ}_{t_{12}},G_{12}\right)}^{\gamma }\right) & \ldots & \left({\left({ℜ}_{r_{1\overset{ˇ}{m}}},{§}_{1\overset{ˇ}{m}}\right)}^{\gamma },{\left({ℜ}_{t_{1\overset{ˇ}{m}}},G_{1\overset{ˇ}{m}}\right)}^{\gamma }\right)\\ \left({\left({ℜ}_{r_{21}},{§}_{21}\right)}^{\gamma },{\left({ℜ}_{t_{21}},G_{21}\right)}^{\gamma }\right) & \left({\left({ℜ}_{r_{22}},{§}_{22}\right)}^{\gamma },{\left({ℜ}_{t_{22}},G_{22}\right)}^{\gamma }\right) & \ldots & \left({\left({ℜ}_{r_{2\overset{ˇ}{m}}},{§}_{2\overset{ˇ}{m}}\right)}^{\gamma },{\left({ℜ}_{t_{2\overset{ˇ}{m}}},G_{2\overset{ˇ}{m}}\right)}^{\gamma }\right)\\ \vdots & \vdots & \ddots & \vdots \\ \left({\left({ℜ}_{r_{\overset{ˇ}{n}1}},{§}_{\overset{ˇ}{n}1}\right)}^{\gamma },{\left({ℜ}_{t_{\overset{ˇ}{n}1}},G_{\overset{ˇ}{n}1}\right)}^{\gamma }\right) & \left({\left({ℜ}_{r_{\overset{ˇ}{n}2}},{§}_{\overset{ˇ}{n}2}\right)}^{\gamma },{\left({ℜ}_{t_{\overset{ˇ}{n}2}},G_{\overset{ˇ}{n}2}\right)}^{\gamma }\right) & \ldots & \left({\left({ℜ}_{r_{\overset{ˇ}{n}\overset{ˇ}{m}}},{§}_{\overset{ˇ}{n}\overset{ˇ}{m}}\right)}^{\gamma },{\left({ℜ}_{t_{\overset{ˇ}{n}\overset{ˇ}{m}}},G_{\overset{ˇ}{n}\overset{ˇ}{m}}\right)}^{\gamma }\right) \end{array}\right]}_{\overset{ˇ}{m}\times \overset{ˇ}{n}} \end{array}$$

**Step 2** Evaluate the support degree $ℸ\left(W_{\psi \phi }^{\gamma },W_{\psi \phi }^{\varepsilon }\right)$ by utilizing Equation (18):

(18) $$\begin{array}{l} ℸ(W_{\psi \phi }^{\gamma },W_{\psi \phi }^{\varepsilon })\\ =1-d(W_{\psi \phi }^{\gamma },W_{\psi \phi }^{\varepsilon })(\gamma ,d=1,2,\ldots ,e;\gamma \neq \varepsilon ), \end{array}$$

where $d\left(W_{\psi \phi }^{\gamma },W_{\psi \phi }^{\varepsilon }\right)$ denotes to indicate the normalized Hamming distance,estimated by Equation (4), between $W_{\psi \phi }^{\gamma }$ and $W_{\psi \phi }^{\varepsilon }$.
**Step 3** The synthesis support matrices ought to have been computed ${\left[ℶ\left(W_{\psi \phi }^{\gamma }\right)\right]}_{\overset{ˇ}{m}\times ň}$ by using Equation (19).

(19) $$\begin{array}{l} ℶ\left(W_{\psi \phi }^{\gamma }\right)=\overset{𝔢}{\underset{\gamma =1;\varepsilon \neq \gamma }{\sum }}ℸ\left(W_{\psi \phi }^{\gamma },W_{\psi \phi }^{\varepsilon }\right) \end{array}$$

**Step 4** The comprehensive power weight matrices should be computed ${\left[{\overset{ˇ}{ð}}_{\psi \phi }^{\gamma }\right]}_{\overset{ˇ}{m}\times \overset{ˇ}{n}}(\gamma =1,2,\ldots ,e)$ using Equation (20).

(20) $${\overset{ˇ}{ð}}_{\psi \phi }^{\gamma }=\frac{\beta^{\prime }\left(1+ℶ(W_{\psi \phi }^{\gamma })\right)}{\sum_{\gamma =1}^{e}\beta^{\prime }\left(1+ℶ(W_{\psi \phi }^{\gamma })\right)}$$

**Step 5** Individual decision matrices are combined. With the support of the 2TL*q*-ROFSSWPA operator, where

(21) $$\begin{array}{l} \text{2TL}q\text{-ROFSSWPA}(W_{1},W_{2},\ldots ,W_{ň})\\ =\left(\begin{array}{l} \Delta \left(\tau {\left(1-{\left(\sum_{\phi =1}^{ň}{\overset{ˇ}{ð}}_{\phi }{\left(1-{\left(\frac{\Delta^{-1}({ℜ}_{r_{\phi }},{§}_{\phi })}{\tau }\right)}^{q}\right)}^{\xi }\right)}^{\frac{1}{\xi }}\right)}^{\frac{1}{q}}\right),\\ \Delta \left(\tau {\left(\sum_{\phi =1}^{ň}{\overset{ˇ}{ð}}_{\phi }{\left(\frac{\Delta^{-1}({ℜ}_{t_{\phi }},G_{\phi })}{\tau }\right)}^{q\xi }\right)}^{\frac{1}{q\xi }}\right) \end{array}\right). \end{array}$$

### 4.1 A method of group decision-making for 2TL*q*-ROFSs based on weighted exponential entropy measure

**Step 6** As a first step, we remove ambiguity in the attributes units by normalizing the values of the attributes in the decision matrix. Equation (22) is used to derive the normalized criterion values.

(22) $$\nabla_{\psi \phi }=\frac{\Theta_{\psi \phi }}{\sum_{\psi =1}^{ň}\Theta_{\psi \phi }},$$

where $\nabla_{\psi \phi }$ is the values of the ϕ*th* criteria for the ψ*th* alternative which have been normalized (unit-less).
**Step 7** Second, we utilize the Equation (23) to figure out the degree of entropy “$E_{\phi }$” for each criterion $\left(\phi =1,2,\ldots ,ň;0\leq E_{\phi }\leq 1\right)$.

(23) $${ℰ}_{\phi }=-\frac{1}{lnn}\sum_{\psi =1}^{ň}\nabla_{\psi \phi }ln(\nabla_{\psi \phi })$$

**Step 8** Thirdly, we calculate the degree of differences (𝔡_*ϕ*_)in each criterion as follows:

(24) $$\begin{array}{l} {𝔡}_{\phi }=1-E_{\phi } \end{array}$$

**Step 9** Lastly, the normalized value 𝔡_*ϕ*_ of the Entropy weight “*β*_*ϕ*_” of the criterion (*ϕ*) can be calculated as follows:

(25) $$\begin{array}{l} \beta_{\phi }=\frac{{𝔡}_{\phi }}{\overset{ň}{\underset{\phi =1}{\sum }}{𝔡}_{\phi }}, \end{array}$$

it should be noted that $\overset{ň}{\underset{\phi =1}{\sum }}\beta_{\phi }=1,\phi =1,2,\ldots ,ň$.

### 4.2 The 2TL*q*-ROF-VIKOR multiple criteria analysis approach

**Step 10** Based on the thorough 2TL*q*-ROF evaluation matrix, to determine the positive ideal solution (PIS) $\hbar_{\phi }^{+}$ and negative ideal solution (NIS) $\hbar_{\phi }^{-}$ for each attribute, utilize the following equations.
The $\hbar_{\phi }^{+}$ and $\hbar_{\phi }^{-}$ indices are calculated from Equation (26) for the positive attributes.

(26) $$\begin{array}{r} \{\begin{array}{c} \hbar_{\phi }^{+}=(\underset{\phi }{\max}({ℜ}_{r_{\psi \phi }},{§}_{\psi \phi }),\underset{\phi }{\min}({ℜ}_{t_{\psi \phi }},G_{\psi \phi }))\\ \hbar_{\phi }^{-}=(\underset{\phi }{\min}({ℜ}_{r_{\psi \phi }},{§}_{\psi \phi }),\underset{\phi }{\max}({ℜ}_{t_{\psi \phi }},G_{\psi \phi })) \end{array};\\ \psi =1,2,\ldots ,\overset{ˇ}{m},\phi =1,2,\ldots ,\overset{ˇ}{n} \end{array}$$

The $\hbar_{\phi }^{+}$ and $\hbar_{\phi }^{-}$ indices are calculated from Equation (27) for the negative attributes.

(27) $$\begin{array}{r} \{\begin{array}{c} \hbar_{\phi }^{+}=(\underset{\phi }{\min}({ℜ}_{r_{\psi \phi }},{§}_{\psi \phi }),\underset{\phi }{\max}({ℜ}_{t_{\psi \phi }},G_{\psi \phi }))\\ \hbar_{\phi }^{-}=(\underset{\phi }{\max}({ℜ}_{r_{\psi \phi }},{§}_{\psi \phi }),\underset{\phi }{\min}({ℜ}_{t_{\psi \phi }},G_{\psi \phi })) \end{array};\\ \psi =1,2,\ldots ,\overset{ˇ}{m},\phi =1,2,\ldots ,\overset{ˇ}{n} \end{array}$$

**Step 11** Next 2TL*q*-ROFS group benefit value $R_{\psi }$ and individual regret value 𝔏_*ψ*_ for every evaluation alternative could be estimated through the Equations (28) and (29):

(28) $$\begin{array}{l} R_{\psi }=\overset{n}{\underset{\phi =1}{\sum }}\beta_{\phi }\frac{\left(\hbar_{\phi }^{+}-\hbar_{\psi \phi }\right)}{\left(\hbar_{\phi }^{+}-\hbar_{\phi }^{-}\right)};\psi =1,2,\ldots ,\overset{ˇ}{m} \end{array}$$

(29) $$\begin{array}{l} L_{\psi }=\underset{\phi }{\max}\left[\beta_{\phi }\frac{\left(\hbar_{\phi }^{+}-\hbar_{\psi \phi }\right)}{\left(\hbar_{\phi }^{+}-\hbar_{\phi }^{-}\right)}\right];\\ \;\psi =1,2,\ldots ,\overset{ˇ}{m},\phi =1,2,\ldots ,\overset{ˇ}{n} \end{array}$$

where $R_{\psi }$ and 𝔏_*ψ*_ identify the group utility and individual regret measures for every alternative. The lower the value of $K_{\psi }$, the better the group utility of the alternative. The opposite is also true the lower the value of 𝔏_ψ_, the lower the individual regret. where the *ϕ*th criteria's weight value is *β*_*ϕ*_.
**Step 12** Equation (30) demonstrates the calculation of the compromise sort index value for each alternative.

(30) $$\begin{array}{c} \Lambda_{\psi }=u\times \left[\frac{{ℛ}_{\psi }-{ℛ}^{+}}{{ℛ}^{-}-{ℛ}^{+}}\right]+\left(1-u\right)\times \left[\frac{L_{\psi }-L^{+}}{L^{-}-L^{+}}\right] \end{array}$$

where ${ℛ}^{+}=\underset{\psi }{\min}{ℛ}_{\psi },\;{ℛ}^{-}=\underset{\psi }{\max}{ℛ}_{\psi },\;L^{+}=\underset{\psi }{\min}L_{\psi },\;L^{-}=\underset{\psi }{\max}L_{\psi }$. And 𝔲 denotes the compromise sorting coefficient, which is commonly assumed to be 0.5.
**Step 13** Ultimately, rank the alternatives based on the acquired values of Λ_ψ_ $\left(\psi =1,2,\ldots ,\overset{ˇ}{m}\right)$, where a higher Λ_ψ_ indicates a less favorable alternative, while a lower value signifies a more favorable one.

**Figure 3:**
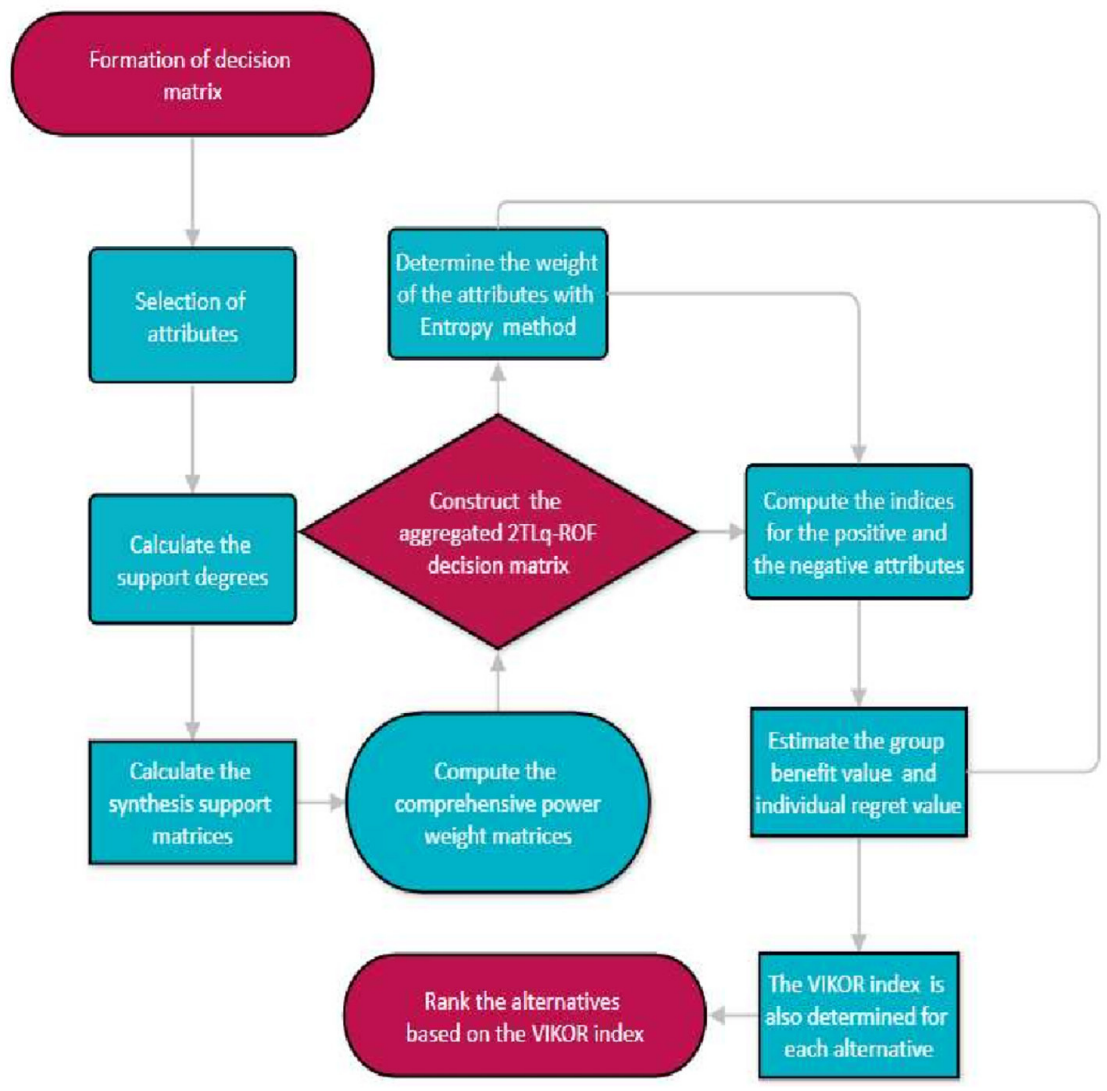
A visual outline of the methodology.

A visual depiction of methodology is given in Figure 3.

## 5 Numerical example

The following subsection presents a numerical illustration to show how flexible and effective the suggested approach is. We choose the most noteworthy instances of artificial intelligence's impact on education in order to validate our work.

### 5.1 Case overview

The teaching, learning, evaluation, and administration of education are all being transformed by AI. AI can improve education quality and effectiveness by offering individualized and adaptable learning experiences, intelligent tutoring systems, automated grading and feedback, and data-driven insights for educators and policymakers. AI can encourage critical thinking, creativity, and teamwork in both students and teachers. However, AI also presents a number of hazards and difficulties for education, including possible ethical, social, and legal repercussions, problems with fairness and the digital divide, and issues with human-AI interaction and trust. As an outcome, it's crucial to ensure that AI is used appropriately in education, for the good of all parties involved, and in a way that respects human dignity and values. Some of the alternatives of the impact of AI in education are:

1. **Ethical analysis(Θ_1_)**
  This focuses on the moral ideals and concepts that underpin the design, creation, and use of AI in education. Regarding issues like privacy, fairness, responsibility, and openness, it also takes into account the moral conundrums and difficulties that may result from the use of AI in education.
2. **Pedagogical analysis** **(Θ_2_)**
  This looks at the ideas and methods of pedagogy that underpin the application of AI in education. Additionally, it assesses how effectively teaching and learning are improved by AI, as well as how AI affects learning in terms of cognitive, emotional, and social elements.
3. **Sociocultural analysis** **(Θ_3_)**
  This looks at the social and cultural implications of artificial intelligence in education. Additionally, it examines how AI may affect the diversity, inclusiveness, and equality of educational opportunities and results, as well as how AI may impact kids' and teachers' cultural identities and values.
4. **Political analysis(Θ_4_)**
  This looks into the interests and power structures that influence the creation and application of AI in education. It also looks at the impact of AI on educational governance, policy, and regulation, as well as the possible drawbacks and upsides of AI for social justice and democracy.
5. **Economic analysis(Θ_5_)**
  This weighs the costs and advantages of artificial intelligence in education. Additionally, it assesses how AI will affect educational institutions' creativity, productivity, and efficiency as well as how AI will affect students' and teachers' capacity to grow their talents and find employment.
6. **Ecological analysis** **(Θ_6_)**
  This focuses on how AI in education affects the environment. It also takes into account the resilience and sustainability of educational methods and solutions enhanced by AI, as well as the potential role of AI in raising awareness of and promoting environmental action.

AI in education can be seen as a MAGDM problem, where multiple stakeholders with different preferences and objectives need to make decisions about the design, implementation, and evaluation of AI systems in education. Based on the previously discussed survey and analysis, we intend to utilize the 2TL*q*-ROF-Entropy-VIKOR method proposed in this article to assess the impact of AI in education. In this context, we will evaluate six alternatives denoted as Θ = {Θ_1_, Θ_2_, …, Θ_6_} with the input of five DMs represented as Φ = {Φ_1_, Φ_2_, Φ_3_, Φ_4_, Φ_5_}, each assigned weights in accordance with β′ = (0.2192, 0.2134, 0.1930, 0.1906, 0.1838)^*T*^ to address the specific problem at hand. These five DMs will select the best alternative according to the assessment of four attributes Ω = {Ω_1_, Ω_2_, Ω_3_, Ω_4_}, as outlined in Table 2. To quantitatively assess each LTS *S*^9^={${ℜ}_{0}^{9}$ : extremely unfavorable$,{ℜ}_{1}^{9}$ : very unfavorable, ${ℜ}_{2}^{9}$: unfavorable, ${ℜ}_{3}^{9}$ : slightly unfavorable, ${ℜ}_{4}^{9}$ : neutral, ${ℜ}_{5}^{9}$ : slightly favorable, ${ℜ}_{6}^{9}$: favorable, ${ℜ}_{7}^{9}$ : very favorable, ${ℜ}_{8}^{9}$ : extremely favorable} five DMs convey their unique viewpoints. The decision matrix, shown in Table 3, summarizes the evaluation values these DMs assigned for each attribute of each alternative.

**Table 2:** Concise overview of assessment attributes.

**Attributes**	**Overview**
Personalization (Ω_1_)	By taking into account students' abilities, preferences, and goals, AI can assist in developing tailored learning paths for them. Additionally, AI has the ability to customize instruction, feedback, and advice for students and teachers to fit their learning preferences and environments.
Automation (Ω_2_)	Tasks like grading, testing, scheduling, and administration that are monotonous, time-consuming, or boring for teachers and students can be automated by AI. By giving teachers data and insights to enhance their teaching methods and student outcomes, AI can also support the function of teachers in the classroom.
Innovation (Ω_3_)	By enabling new forms of learning like gamification, simulation, virtual reality, and augmented reality, AI can promote innovation and creativity in education. By fostering global collaboration, communication, and interaction between students, professors, and specialists, AI can also improve the quality and accessibility of education.
Ethics (Ω_4_)	Concerning issues like privacy, security, prejudice, accountability, and transparency, AI also presents certain ethical problems and risks for education. To guarantee that AI is in line with the principles and objectives of education and upholds the rights and dignity of both students and teachers, rigorous planning, implementation, and evaluation are necessary.

**Table 3:** Decision matrices by DMs utilizing the 2TL*q*-ROFNs.

		Ω_1_	Ω_2_	Ω_3_	Ω_4_
	Θ_1_	((ℜ_7_, 0), (ℜ_6_, 0))	((ℜ_3_, 0), (ℜ_7_, 0))	((ℜ_7_, 0), (ℜ_5_, 0))	((ℜ_1_, 0), (ℜ_2_, 0))
	Θ_2_	((ℜ_5_, 0), (ℜ_3_, 0))	((ℜ_5_, 0), (ℜ_1_, 0))	((ℜ_3_, 0), (ℜ_6_, 0))	((ℜ_4_, 0), (ℜ_7_, 0))
Φ_1_	Θ_3_	((ℜ_4_, 0), (ℜ_7_, 0))	((ℜ_4_, 0), (ℜ_3_, 0))	((ℜ_1_, 0), (ℜ_2_, 0))	((ℜ_5_, 0), (ℜ_4_, 0))
	Θ_4_	((ℜ_6_, 0), (ℜ_2_, 0))	((ℜ_4_, 0), (ℜ_1_, 0))	((ℜ_4_, 0), (ℜ_5_, 0))	((ℜ_7_, 0), (ℜ_6_, 0))
	Θ_5_	((ℜ_3_, 0), (ℜ_1_, 0))	((ℜ_3_, 0), (ℜ_4_, 0))	((ℜ_2_, 0), (ℜ_6_, 0))	((ℜ_4_, 0), (ℜ_1_, 0))
	Θ_6_	((ℜ_1_, 0), (ℜ_6_, 0))	((ℜ_2_, 0), (ℜ_7_, 0))	((ℜ_1_, 0), (ℜ_7_, 0))	((ℜ_3_, 0), (ℜ_7_, 0))
	Θ_1_	((ℜ_7_, 0), (ℜ_5_, 0))	((ℜ_2_, 0), (ℜ_5_, 0))	((ℜ_4_, 0), (ℜ_6_, 0))	((ℜ_1_, 0), (ℜ_7_, 0))
	Θ_2_	((ℜ_5_, 0), (ℜ_6_, 0))	((ℜ_7_, 0), (ℜ_2_, 0))	((ℜ_1_, 0), (ℜ_2_, 0))	((ℜ_4_, 0), (ℜ_7_, 0))
Φ_2_	Θ_3_	((ℜ_4_, 0), (ℜ_7_, 0))	((ℜ_2_, 0), (ℜ_6_, 0))	((ℜ_7_, 0), (ℜ_3_, 0))	((ℜ_3_, 0), (ℜ_5_, 0))
	Θ_4_	((ℜ_1_, 0), (ℜ_2_, 0))	((ℜ_4_, 0), (ℜ_3_, 0))	((ℜ_5_, 0), (ℜ_5_, 0))	((ℜ_3_, 0), (ℜ_4_, 0))
	Θ_5_	((ℜ_5_, 0), (ℜ_1_, 0))	((ℜ_1_, 0), (ℜ_7_, 0))	((ℜ_4_, 0), (ℜ_3_, 0))	((ℜ_2_, 0), (ℜ_5_, 0))
	Θ_6_	((ℜ_2_, 0), (ℜ_6_, 0))	((ℜ_3_, 0), (ℜ_4_, 0))	((ℜ_6_, 0), (ℜ_2_, 0))	((ℜ_6_, 0), (ℜ_1_, 0))
	Θ_1_	((ℜ_1_, 0), (ℜ_6_, 0))	((ℜ_5_, 0), (ℜ_1_, 0))	((ℜ_4_, 0), (ℜ_3_, 0))	((ℜ_2_, 0), (ℜ_2_, 0))
	Θ_2_	((ℜ_3_, 0), (ℜ_2_, 0))	((ℜ_1_, 0), (ℜ_3_, 0))	((ℜ_3_, 0), (ℜ_4_, 0))	((ℜ_4_, 0), (ℜ_1_, 0))
Φ_3_	Θ_3_	((ℜ_2_, 0), (ℜ_4_, 0))	((ℜ_3_, 0), (ℜ_3_, 0))	((ℜ_2_, 0), (ℜ_5_, 0))	((ℜ_5_, 0), (ℜ_1_, 0))
	Θ_4_	((ℜ_1_, 0), (ℜ_5_, 0))	((ℜ_6_, 0), (ℜ_1_, 0))	((ℜ_1_, 0), (ℜ_6_, 0))	((ℜ_3_, 0), (ℜ_1_, 0))
	Θ_5_	((ℜ_4_, 0), (ℜ_3_, 0))	((ℜ_5_, 0), (ℜ_2_, 0))	((ℜ_4_, 0), (ℜ_1_, 0))	((ℜ_2_, 0), (ℜ_4_, 0))
	Θ_6_	((ℜ_2_, 0), (ℜ_5_, 0))	((ℜ_2_, 0), (ℜ_1_, 0))	((ℜ_4_, 0), (ℜ_2_, 0))	((ℜ_1_, 0), (ℜ_5_, 0))
	Θ_1_	((ℜ_2_, 0), (ℜ_5_, 0))	((ℜ_3_, 0), (ℜ_7_, 0))	((ℜ_4_, 0), (ℜ_6_, 0))	((ℜ_2_, 0), (ℜ_4_, 0))
	Θ_2_	((ℜ_3_, 0), (ℜ_3_, 0))	((ℜ_5_, 0), (ℜ_6_, 0))	((ℜ_5_, 0), (ℜ_5_, 0))	((ℜ_7_, 0), (ℜ_2_, 0))
Φ_4_	Θ_3_	((ℜ_5_, 0), (ℜ_3_, 0))	((ℜ_1_, 0), (ℜ_3_, 0))	((ℜ_1_, 0), (ℜ_6_, 0))	((ℜ_2_, 0), (ℜ_4_, 0))
	Θ_4_	((ℜ_6_, 0), (ℜ_1_, 0))	((ℜ_7_, 0), (ℜ_1_, 0))	((ℜ_3_, 0), (ℜ_6_, 0))	((ℜ_1_, 0), (ℜ_7_, 0))
	Θ_5_	((ℜ_1_, 0), (ℜ_4_, 0))	((ℜ_4_, 0), (ℜ_5_, 0))	((ℜ_2_, 0), (ℜ_5_, 0))	((ℜ_4_, 0), (ℜ_3_, 0))
	Θ_6_	((ℜ_7_, 0), (ℜ_2_, 0))	((ℜ_2_, 0), (ℜ_4_, 0))	((ℜ_6_, 0), (ℜ_3_, 0))	((ℜ_5_, 0), (ℜ_1_, 0))
	Θ_1_	((ℜ_1_, 0), (ℜ_2_, 0))	((ℜ_5_, 0), (ℜ_3_, 0))	((ℜ_1_, 0), (ℜ_7_, 0))	((ℜ_7_, 0), (ℜ_2_, 0))
	Θ_2_	((ℜ_2_, 0), (ℜ_4_, 0))	((ℜ_6_, 0), (ℜ_3_, 0))	((ℜ_3_, 0), (ℜ_5_, 0))	((ℜ_4_, 0), (ℜ_4_, 0))
Φ_5_	Θ_3_	((ℜ_3_, 0), (ℜ_6_, 0))	((ℜ_7_, 0), (ℜ_4_, 0))	((ℜ_1_, 0), (ℜ_6_, 0))	((ℜ_1_, 0), (ℜ_7_, 0))
	Θ_4_	((ℜ_1_, 0), (ℜ_4_, 0))	((ℜ_5_, 0), (ℜ_2_, 0))	((ℜ_4_, 0), (ℜ_7_, 0))	((ℜ_5_, 0), (ℜ_6_, 0))
	Θ_5_	((ℜ_5_, 0), (ℜ_3_, 0))	((ℜ_4_, 0), (ℜ_7_, 0))	((ℜ_5_, 0), (ℜ_7_, 0))	((ℜ_5_, 0), (ℜ_3_, 0))
	Θ_6_	((ℜ_4_, 0), (ℜ_6_, 0))	((ℜ_1_, 0), (ℜ_6_, 0))	((ℜ_2_, 0), (ℜ_4_, 0))	((ℜ_2_, 0), (ℜ_5_, 0))

### 5.2 The results of the suggested method

This subsection assesses the suitability of an innovative method for decision-making contingent on the amalgamation of the VIKOR and Entropy methodologies with 2TL*q*-ROF data to determine how well it analyzes the impact of AI on education. The procedures to be performed in the procedure are listed below:

**Step 1**. We construct the 2TL*q*-ROF evaluation matrix $\hbar^{\gamma }={\left[W_{\psi \phi }^{\gamma }\right]}_{6\times 4}={\left(({ℜ}_{r_{\psi \phi }}^{\gamma },{§}_{\psi \phi }^{\gamma }),({ℜ}_{t_{\psi \phi }}^{\gamma },G_{\psi \phi }^{\gamma })\right)}_{6\times 4}(\psi =1,2,3,\ldots ,6,\phi =1,2,3,4)$ and *γ* = 1, 2, 3, 4, 5), subsequently provides the assessments of five DMs as established in Table 3.
**Step 2**. Calculated using Equation (28), we have the support $ℸ(W_{\psi \phi }^{\gamma },W_{\psi \phi }^{\varepsilon })(\psi =1,2,\ldots ,6;\;\phi =1,2,3,4;\;\gamma =1,2,3,4,5;\;\varepsilon =1,2,3,4,5$ and γ≠ε). For convenience, we present $ℸ\left(W_{\psi \phi }^{\gamma },W_{\psi \phi }^{\varepsilon }\right)$ by ℸ^γε^(γ = 1, 2, 3, 4, 5; ε = 1, 2, 3, 4, 5 and γ≠ε). Consequently, we can obtain:

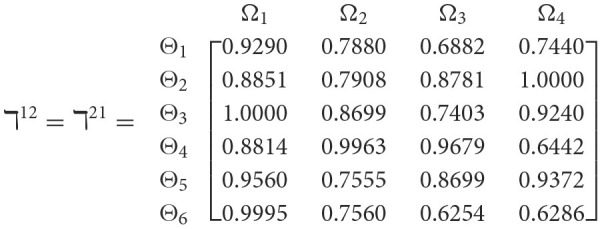



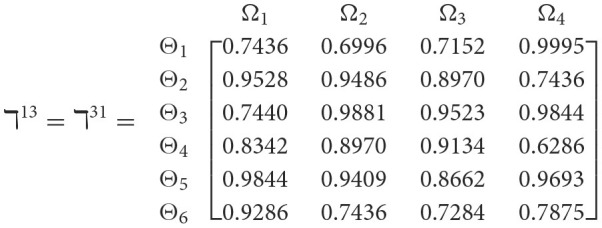



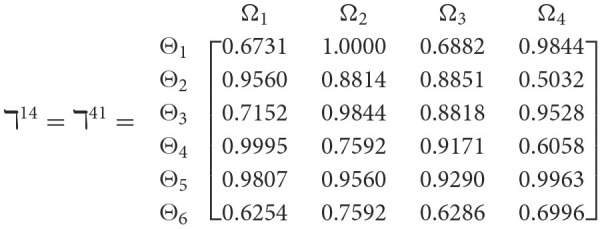



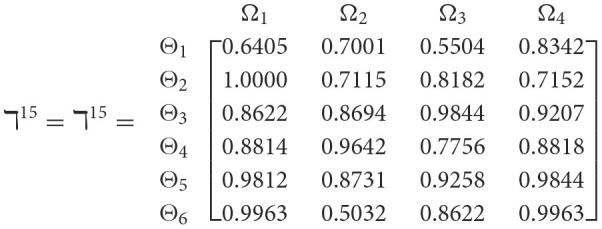



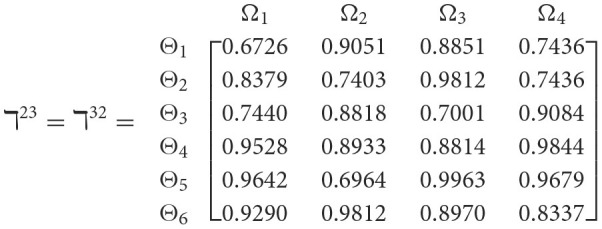



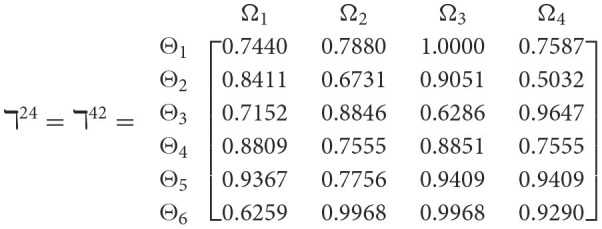



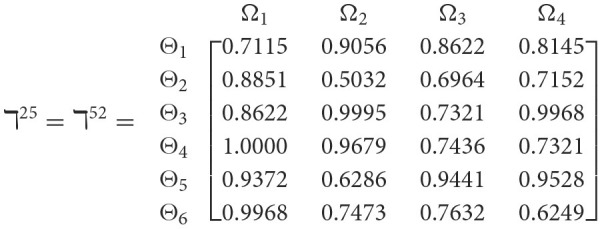



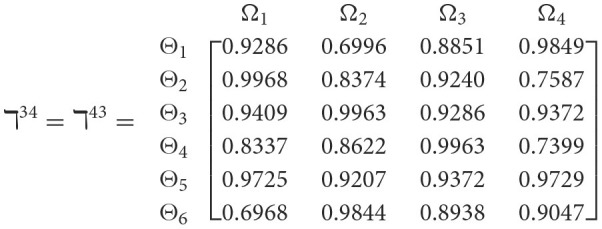



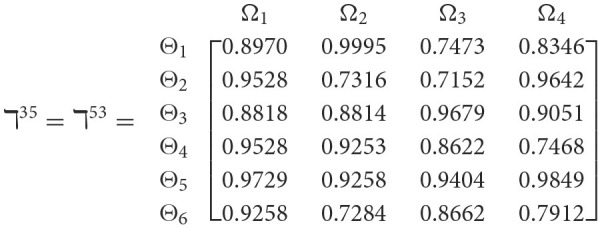



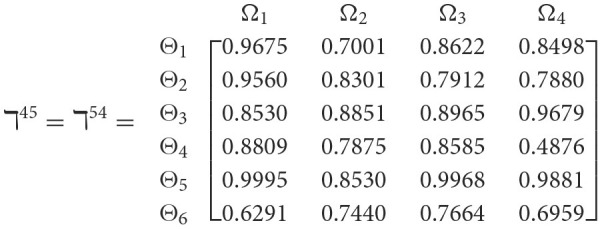

**Step 3**. We have the overall support matrices $ℶ\left(W_{\psi \phi }^{\gamma }\right)$ of the 2TL*q*-ROFN that correspond with Equation (29). For convenience, we depict the values $ℶ\left(W_{\psi \phi }^{\gamma }\right)\left(\psi =1,2,\ldots ,6;\;\phi =1,2,3,4;\;\gamma =1,2,3,4,5\right)$ as a matrix ℶ^*γ*^ (γ = 1, 2, 3, 4, 5), illustrated below:

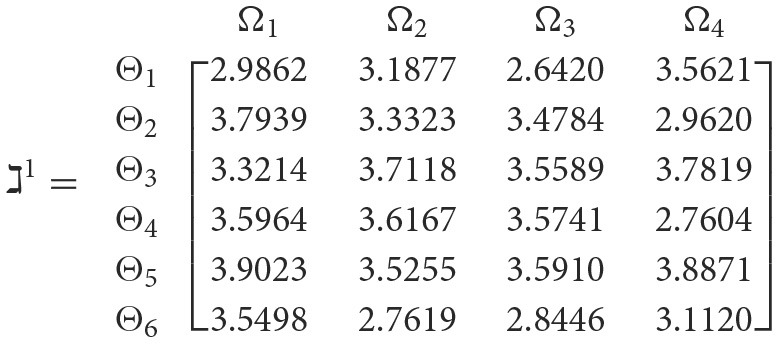



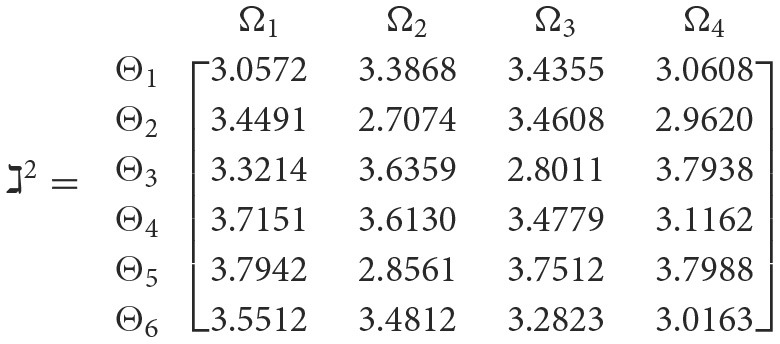



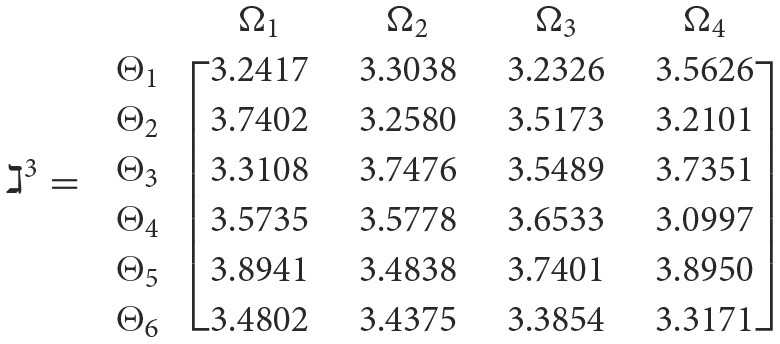



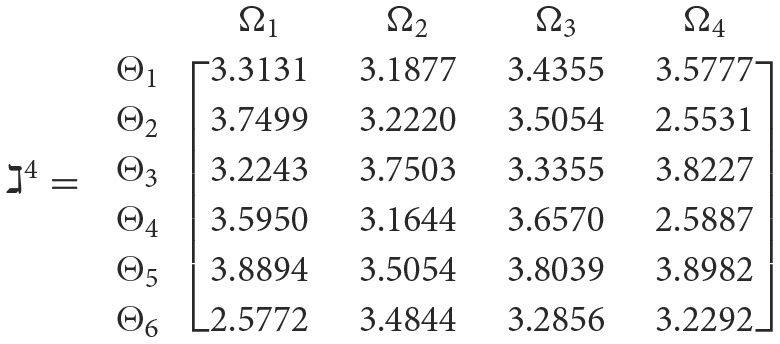



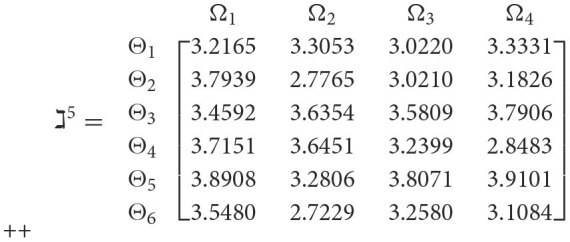


++

**Step 4**. We utilize the weighted power matrix of the judgment decision maker connected to the 2TL*q*-ROFN in accordance with Equation (26). The values $ð\left(W_{\psi \phi }^{\gamma }\right)\left(\psi =1,2,\ldots ,6;\;\phi =1,2,3,4;\;\gamma =1,2,3,4,5\right)$ are expressed by the matrix *ð*^γ^ (γ = 1, 2, 3, 4, 5), which is depicted as listed below:

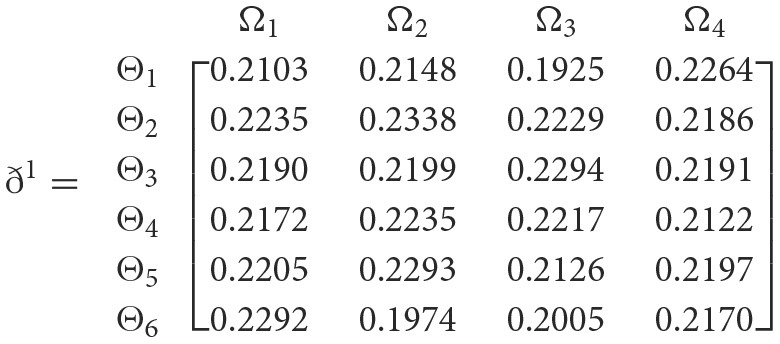



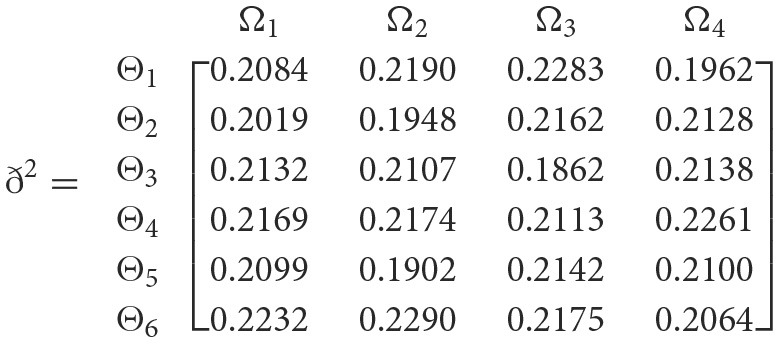



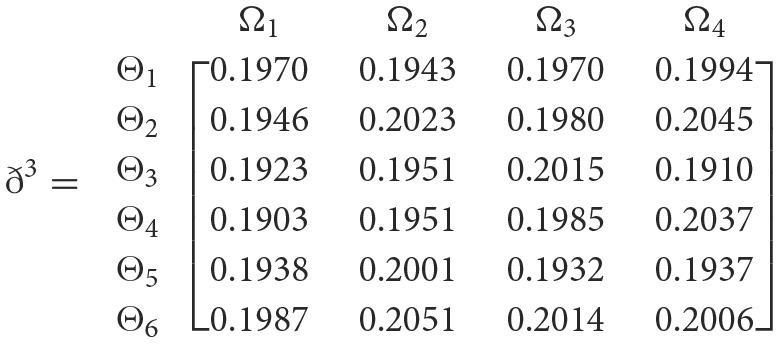



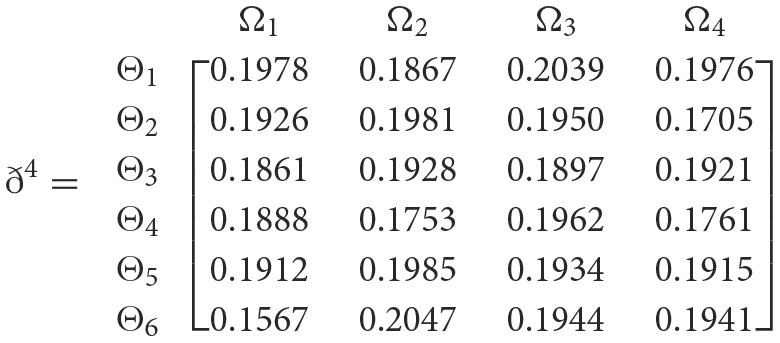



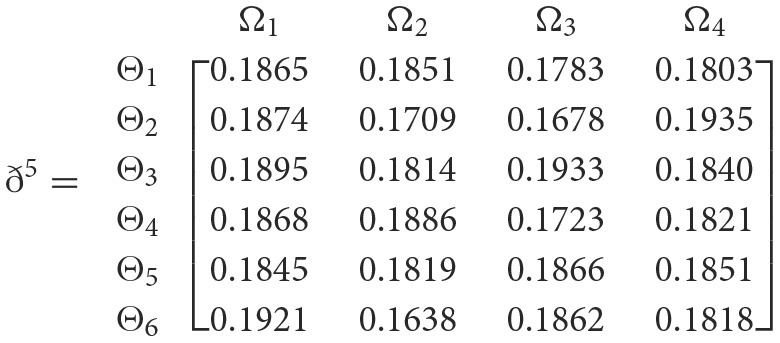

**Step 5**. The 2TL*q*-ROFSSWPA aggregating operator by Equation (21) suggests utilizing overall $\left[W_{\psi \phi }^{\gamma }\right]$ to $\left[W_{\psi \phi }\right]$. Table 4 depicts fused 2TL*q*-ROFNs matrix $\hbar ={\left[W_{\psi \phi }\right]}_{\hat{m}\times ň}$(Suppose *q* = 5, ξ = −2, τ=8, and β′ = (0.2192, 0.2134, 0.1930, 0.1906, 0.1838)^*T*^)
**Step 6**. Normalize the decision matrix's attribute values by utilizing Equation (22) as shown below.

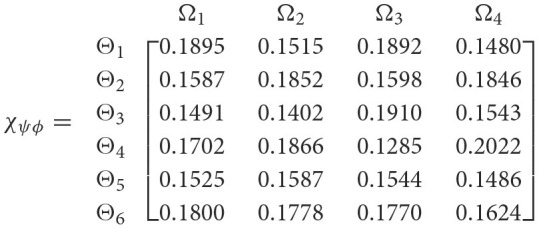

**Step 7**. In consideration of the Equation (23), degree of the Entropy $E_{\phi }$ for Ω_*ϕ*_(*ϕ* = 1, 2, 3, 4), which result is displayed in Table 5.
**Step 8**. In consideration of the Equation (24), the degree of differences 𝔡_*ϕ*_ for Ω_*ϕ*_(*ϕ* = 1, 2, 3, 4), subsequently is displayed in Table 5.
**Step 9**. In regard to the Equation (25), the Entropy weights *β*_*ϕ*_ for Ω_*ϕ*_(*ϕ* = 1, 2, 3, 4), which is shown in Table 5.
**Step 10**. In this stage, the VIKOR method's concept is used to assess each alternative's anticipated effect on education using AI. Compute PIS and NIS utilizing the Equations (26) and (27) are displayed in Table 6.
**Step 11**. Based on the vales of ℏ^+^ and ℏ^−^ and β by using Equations (28) and (29) estimate the $R_{\psi }$ and 𝔏_ψ_. The computation's findings $R_{\psi }$ and 𝔏_ψ_ for six alternatives are listed in Table 7.
**Step 12**. Calculate the VIKOR index for every alternative according to Equation (30) are shown in the Table 8:
**Step 13**. Corresponding to the VIKOR index, order possible alternatives.

$$\Theta_{2}≻\Theta_{6}≻\Theta_{1}≻\Theta_{3}≻\Theta_{4}≻\Theta_{5}$$

Therefore, Θ_2_ is the best alternative.

**Table 4:** The fused 2TL*q*-ROF decision matrix by the 2TL*q*-ROFSSWPA operator.

**Comprehensive decision matrix**		
**Alternatives**	Ω_1_	Ω_2_
Θ_1_	((ℜ_6_, 0.4743), (ℜ_5_, −0.3810))	((ℜ_4_, 0.2531), (ℜ_1_, 0.1779))
Θ_2_	((ℜ_5_, −0.3810), (ℜ_2_, 0.3434))	((ℜ_6_, 0.0158), (ℜ_1_, 0.1563))
Θ_3_	((ℜ_4_, 0.1776), (ℜ_4_, −0.4712))	((ℜ_3_, 0.1026), (ℜ_3_, 0.1529))
Θ_4_	((ℜ_5_, 0.2371), (ℜ_1_, 0.1810))	((ℜ_6_, 0.0597), (ℜ_1_, 0.0535))
Θ_5_	((ℜ_4_, −0.0304), (ℜ_1_, 0.0879))	((ℜ_5_, −0.1260), (ℜ_2_, 0.3452))
Θ_6_	((ℜ_6_, −0.2882), (ℜ_2_, 0.4072))	((ℜ_6_, −0.2495), (ℜ_1_, 0.1717))
**Alternatives**	Ω_3_	Ω_4_
Θ_1_	((ℜ_6_, −0.0550), (ℜ_4_, −0.4737))	((ℜ_4_, −0.3485), (ℜ_2_, 0.1781))
Θ_2_	((ℜ_4_, 0.2190), (ℜ_2_, 0.3308))	((ℜ_6_, −0.1043), (ℜ_1_, 0.1719))
Θ_3_	((ℜ_6_, −0.1490), (ℜ_2_, 0.3138))	((ℜ_4_, 0.2900), (ℜ_1_, 0.1800))
Θ_4_	((ℜ_4_, −0.0137), (ℜ_5_, 0.3560))	((ℜ_6_, 0.4353), (ℜ_1_, 0.1724))
Θ_5_	((ℜ_3_, 0.4453), (ℜ_1_, 0.1787))	((ℜ_4_, −0.3652), (ℜ_1_, 0.1636))
Θ_6_	((ℜ_5_, 0.2872), (ℜ_2_, 0.1801))	((ℜ_5_, −0.1122), (ℜ_1_, 0.0958))

**Table 5:** Values generated by the entropy algorithm.

Ω_ϕ_	$E_{\phi }$	𝔡_ϕ_	β_ϕ_
Ω_1_	0.9979	0.0021	0.1510
Ω_2_	0.9969	0.0031	0.2218
Ω_3_	0.9951	0.0049	0.3474
Ω_4_	0.9960	0.0040	0.2799

**Table 6:** PIS and NIS values for each attribute.

	Ω_1_	Ω_2_	Ω_3_	Ω_4_
ℏ^+^	((ℜ_6_, 0.4743), (ℜ_5_, −0.3810))	((ℜ_6_, 0.0597), (ℜ_1_, 0.0535))	((ℜ_6_, −0.1490), (ℜ_2_, 0.3138))	((ℜ_6_, 0.4353), (ℜ_1_, 0.1724))
ℏ^−^	((ℜ_4_, 0.1776), (ℜ_4_, −0.4712))	((ℜ_3_, 0.1026), (ℜ_3_, 0.1529))	((ℜ_6_, −0.1490), (ℜ_2_, 0.3138))	((ℜ_4_, −0.3485), (ℜ_2_, 0.1781))

**Table 7:** Computed values of $R_{\psi }$ and 𝔏_ψ_.

	Θ_1_	Θ_2_	Θ_3_	Θ_4_	Θ_5_	Θ_6_
$R_{\psi }$	0.4573	0.3859	0.6201	0.4197	0.7518	0.3608
𝔏_ψ_	0.2799	0.1735	0.2473	0.3474	0.2767	0.2055

**Table 8:** The values of Λ_ψ_ of alternatives.

	Θ_1_	Θ_2_	Θ_3_	Θ_4_	Θ_5_	Θ_6_
Λ_ψ_	0.4293	0.0322	0.5437	0.5753	0.7968	0.0920

### 5.3 Comparative analysis

**Figure 4:**
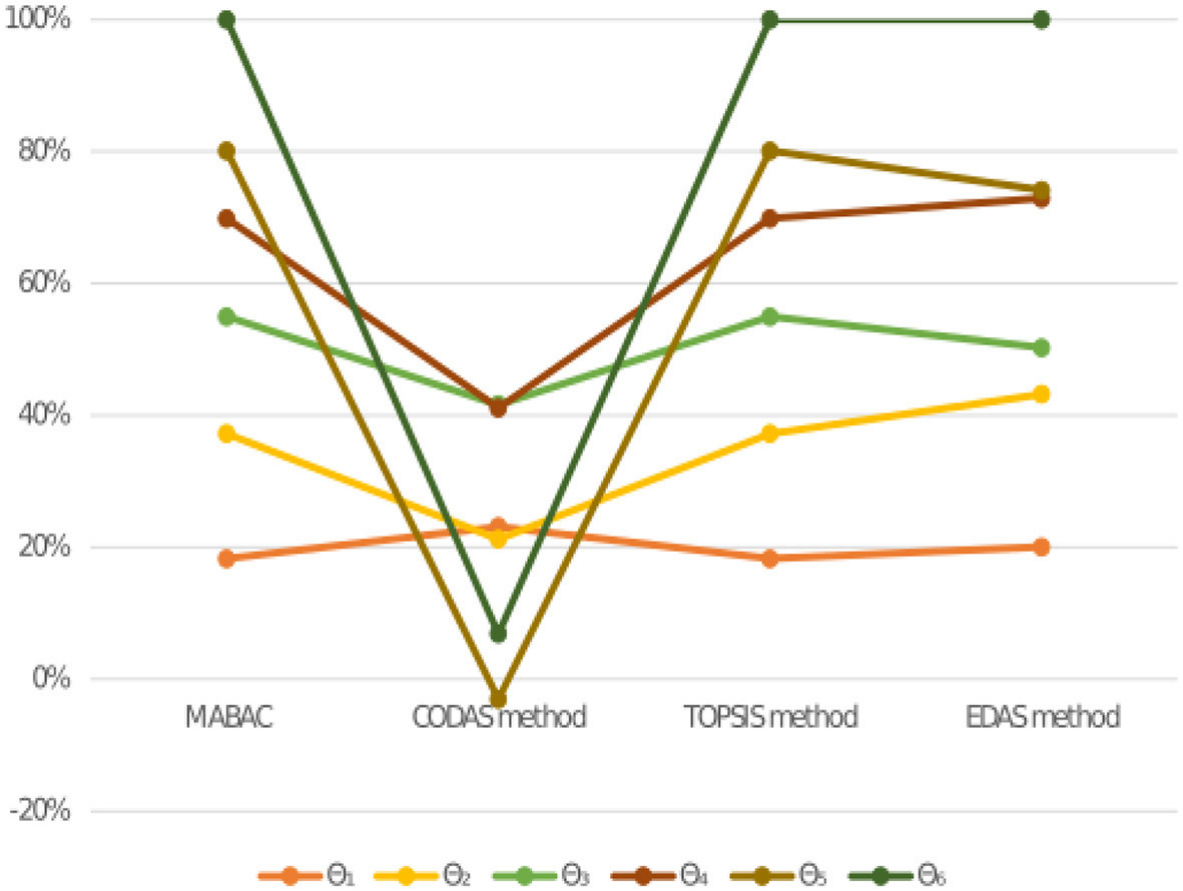
Rank of alternatives utilizing various MAGDM methods.

We conduct a comparative analysis in this section to demonstrate the efficacy and validity of the novel approach used in this research. The numerical and ranking outcomes are listed below in Tables 9, 10 and Figure 2 illustrates the ranking of alternatives using different MAGDM methods. Figure 4 demonstrates alternative rankings with varied MAGDM methods. We compare the suggested approach with several different methods, such as the MABAC method (Naz et al., 2023c), the CODAS method (Naz et al., 2022b), the TOPSIS method (Naz et al., 2023a), and the EDAS method (Naz et al., 2022a). Table 10 exhibits minor variations in judgment order, but it's crucial to emphasize that the top choices consistently differ. To achieve this, we initially compare the proposed approach with the MABAC method, resulting in the ranking Θ_6_>Θ_2_>Θ_1_>Θ_3_>Θ_4_>Θ_5_, with Θ_6_ emerging as the best alternative. Next, we evaluate our proposed approach against the CODAS method, which yields the ranking Θ_1_>Θ_3_>Θ_6_>Θ_4_>Θ_2_>Θ_5_, and identifies Θ_1_ as the best alternative. Following that, we assess the proposed approach against the TOPSIS method, resulting in the ranking Θ_6_>Θ_2_>Θ_1_>Θ_3_>Θ_4_>Θ_5_, where Θ_6_ emerges as the top choice. Finally, our comparison with the EDAS method leads to the ranking Θ_6_>Θ_2_>Θ_4_>Θ_1_>Θ_3_>Θ_5_, with Θ_6_ being identified as the best alternative.

**Table 9:** Comparison of the impact of AI in education with different MAGDM methods by the 2TL*q*-ROFSSPWA operator.

	**Scoring outcome by**	**Scoring outcome by**	**Scoring outcome by**	**Scoring outcome by**
**Alternative**	**MABAC method (Naz et al.**, **2023c****)**	**CODAS method (Naz et al.**, **2022b****)**	**TOPSIS method (Naz et al.**, **2023a****)**	**EDAS method (Naz et al.**, **2022a****)**
Θ_1_	0.5643	0.1261	0.5640	0.5859
Θ_2_	0.5848	−0.0104	0.5850	0.6782
Θ_3_	0.5479	0.1113	0.5473	0.2066
Θ_4_	0.4594	−0.0029	0.4599	0.6623
Θ_5_	0.3165	−0.2406	0.3164	0.0356
Θ_6_	0.6147	0.0540	0.6146	0.7576

**Table 10:** Ranking outcomes by the 2TL*q*-ROFSSPWA operator.

**MAGDM methods**	**Ranking results**	**The best alternative**
The purposed method	Θ_2_>Θ_6_>Θ_1_>Θ_3_>Θ_4_>Θ_5_	Θ_2_
MABAC (Naz et al., 2023c)	Θ_6_>Θ_2_>Θ_1_>Θ_3_>Θ_4_>Θ_5_	Θ_6_
CODAS (Naz et al., 2022b)	Θ_1_>Θ_3_>Θ_6_>Θ_4_>Θ_2_>Θ_5_	Θ_1_
TOPSIS (Naz et al., 2023a)	Θ_6_>Θ_2_>Θ_1_>Θ_3_>Θ_4_>Θ_5_	Θ_6_
EDAS (Naz et al., 2022a)	Θ_6_>Θ_2_>Θ_4_>Θ_1_>Θ_3_>Θ_5_	Θ_6_

In conclusion, the comprehensive comparative analysis of the new approach against established methods yielded intriguing results. The purposed method stands out among the existing MAGDM methods by balancing best and worst performance, Θ_2_ as the best alternative. Its suitability depends on specific decision objectives and criteria importance. Under the MABAC method, Θ_6_ is the optimal choice, showcasing the new approach's effectiveness in specific contexts. Conversely, the CODAS method placed Θ_1_ at the forefront, illustrating its adaptability. The TOPSIS method favored Θ_6_ as the best alternative, highlighting its robustness. Likewise, the EDAS method reinforced Θ_6_ as the optimal choice, underscoring the new approach's versatility. These results emphasize the importance of tailoring the choice of alternative to the evaluation context while recognizing Θ_2_ as a robust choice for a wide range of practical applications of the new approach.

## 6 Conclusion

Teaching, learning, assessment, and administration are just a few of the areas of education that could be drastically changed by AI, a rapidly evolving field. However, there are also many risks and difficulties associated with the adoption and use of AI in education, including moral, legal, and practical concerns. Because of this, it's crucial to assess the impact of AI on education from a variety of angles and using a variety of criteria, as well as to take into account any trade-offs or unpredictability when making decisions. The 2TL*q*-ROFS, also recognized as the generalized version of 2TL and *q*-ROFS, was utilized throughout this study to deal with the ambiguity and imperfection related to group decision-making challenges. This new fuzzy set conveys linguistic information at different levels of granularity and uncertainty while being able to deal with dynamic and complex decision-making contexts. The SSWPA operator is a generalized aggregating operator that can recognize relationships and correlations between decision-makers and other characteristics. The 2TL*q*-ROFSSWPA operator was proposed to combine the ambiguous evaluation findings in order to aggregate the 2TL*q*-ROF information. We also developed the 2TL*q*-ROF-Entropy-VIKOR model, and after combining the traditional Entropy-VIKOR technique with 2TL*q*-ROF data, we gave a thorough explanation of the calculation stages. It has been demonstrated that the suggested model is more accurate and useful because it considers a compromise between personal regret minimization and group utility maximization. Following that, the fundamental ideas and procedures of the suggested 2TL*q*-ROF-Entropy-VIKOR method were discussed. Its significance is as follows: (1) It can minimize the information loss and distortion brought on by the conventional linguistic aggregation operators and fuzzy number ranking methods. (2) It can handle complicated and uncertain decision issues incorporating linguistic and numerical information. (3) With the help of the decision-maker's preferences and the entropy measure, it is possible to determine the subjective and objective weights of the criterion. (4) A rating of all the options is also possible, as is a compromise solution that is the furthest from the negative ideal solution and the closest to the perfect solution. (5) Some of the shortcomings and restrictions of the current techniques, such as the Pythagorean fuzzy VIKOR method, the intuitionistic fuzzy VIKOR method, and the linguistic hesitant fuzzy VIKOR method, can be addressed by it. A group decision-making process using the 2TL*q*-ROF-Entropy-VIKOR model is then utilized to calculate the weights of the attributes finding and the most preferred alternative. In the case study section, to exemplify the implementation of the developed method and emphasize its efficacy, a numerical illustration was presented. Finally, the comparative analysis's findings demonstrated that the established method could be successfully used to handle MAGDM issues in the 2TL*q*-ROF environment. Even if the suggested model is superior in theory and application, this research still has certain drawbacks. The proposed model, for instance, includes a number of parameters, and altering those parameters could produce different outcomes. Future studies will focus on developing the fuzzy behavioral VIKOR approach to the 2TL*q*-ROFS under the reference ideal theory. In follow-up research, we'll use some fresh approaches to further generalize these operators. At the same time, we will broaden the applicability of these AOs to a variety of uncertain fields, such as risk management, the location of electric vehicle charging stations, and many others.

## Data availability statement

The raw data supporting the conclusions of this article will be made available by the authors, without undue reservation.

## Author contributions

AM: Methodology, Supervision, Writing – review & editing. ZU: Software, Supervision, Validation, Writing – review & editing. AO: Investigation, Methodology, Resources, Writing – review & editing. MR: Conceptualization, Methodology, Software, Visualization, Writing – original draft. SE: Formal analysis, Resources, Writing – review & editing. KY: Investigation, Software, Writing – review & editing.
